# Structure-guided mutagenesis of a mucin-selective metalloprotease from *Akkermansia muciniphila* alters substrate preferences

**DOI:** 10.1016/j.jbc.2022.101917

**Published:** 2022-04-09

**Authors:** D. Judy Shon, Daniel Fernandez, Nicholas M. Riley, Michael J. Ferracane, Carolyn R. Bertozzi

**Affiliations:** 1 Department of Chemistry and Stanford ChEM-H, Stanford University, Stanford, California, USA; 2 Stanford ChEM-H Macromolecular Structure Knowledge Center, Stanford University, Stanford, California, USA; 3 Department of Chemistry, University of Redlands, Redlands, California, USA; 4 Howard Hughes Medical Institute, Stanford University, Stanford, California, USA

**Keywords:** X-ray crystallography, enzyme structure, metalloprotease, O-glycosylation, mucin, mucinase, molecular docking, glycoproteomics, microbiome, E:S, enzyme:substrate, FA, formic acid, GuHCl, guanidine hydrochloride, HCD, higher-energy collisional dissociation, MUC2, mucin 2, MUC16, mucin 16, PSGL-1, P-selectin glycoprotein ligand 1, SSRL, Stanford Synchrotron Radiation Lightsource

## Abstract

*Akkermansia muciniphila*, a mucin-degrading microbe found in the human gut, is often associated with positive health outcomes. The abundance of *A. muciniphila* is modulated by the presence and accessibility of nutrients, which can be derived from diet or host glycoproteins. In particular, the ability to degrade host mucins, a class of proteins carrying densely O-glycosylated domains, provides a competitive advantage in the sustained colonization of niche mucosal environments. Although *A. muciniphila* is known to rely on mucins as a carbon and nitrogen source, the enzymatic machinery used by this microbe to process mucins in the gut is not yet fully characterized. Here, we focus on the mucin-selective metalloprotease, Amuc_0627 (AM0627), which is known to cleave between adjacent residues carrying truncated core 1 O-glycans. We showed that this enzyme is capable of degrading purified mucin 2 (MUC2), the major protein component of mucus in the gut. An X-ray crystal structure of AM0627 (1.9 Å resolution) revealed O-glycan–binding residues that are conserved between structurally characterized enzymes from the same family. We further rationalized the substrate cleavage motif using molecular modeling to identify nonconserved glycan-interacting residues. We conclude that mutagenesis of these residues resulted in altered substrate preferences down to the glycan level, providing insight into the structural determinants of O-glycan recognition.

Human health and disease risk are highly influenced by microbial communities. In particular, the gastrointestinal tract is home to trillions of organisms that exist in a mutually beneficial equilibrium with the host. This gut microbiota not only aids in breaking down complex carbohydrates but it also colonizes niche environments that could otherwise be infiltrated by pathogenic organisms (1). Thus, the human immune system has adapted to tolerate this equilibrium state (2). However, environmental and host-related factors can perturb this balance by altering the composition of the microbiota in a condition known as dysbiosis (3). Dysbiosis can change the functions of the gut microbiota and increase the risk for metabolic disorders, inflammatory bowel diseases, and cancer (4, 5, 6). The composition of the microbiota is regulated in part by intestinal mucus layers, which serve as barriers to certain organisms while selecting for others via the presented glycan repertoire (7). The balance between mucus production and breakdown can therefore have an indirect influence on human health.

Mucosal layers throughout the human body are composed of mucins, a class of proteins carrying densely O-glycosylated domains. These domains are characterized by a high frequency of serine and threonine residues modified with an initiating α-GalNAc that is often further elaborated with other monosaccharides to generate highly diverse structures. Mucins additionally consist of variable number of tandem repeat domains, which further expand complexity and heterogeneity (7). As a result, microbial colonization of the mucus layer requires a repertoire of hydrolytic enzymes that include peptidases, sulfatases, and glycoside hydrolases.

Some microbes have evolved the genetic machinery to effectively degrade host mucins for nutrients, allowing colonization of niche mucosal regions that are less accessible to other organisms. One such mucin degrader is *Akkermansia muciniphila*, an abundant Gram-negative bacterium that accounts for 1% to 5% of the adult intestinal microbiota (8, 9). Increased prevalence of *A. muciniphila* has been shown to have a beneficial effect in the context of high-fat diet-induced metabolic disorders (10, 11), inflammatory bowel diseases (12, 13, 14), and response to checkpoint immunotherapy against cancer (15). These observations have spurred attempts to boost the abundance of *A. muciniphila* in the gut via supplementation of mucin-derived O-glycans in mice (16, 17) or administration of the bacteria itself in humans (18). The addition of mucin-type O-glycans promotes colonization by *A. muciniphila* because this microbe relies on mucins as a carbon and nitrogen source (9). Despite these links, the collection of enzymes and precise mechanisms used by *A. muciniphila* to process mucins in the gut are not yet fully characterized. Previous work has shown that in the absence of dietary fiber, the gut microbiota must resort to host mucus glycoproteins for nutrients and will upregulate transcripts encoding glycoside hydrolases and M60-like proteases (19).

The M60-like or Pfam 13402 (PF13402) family consists of hundreds of enzymes from organisms known to inhabit mucosal host environments. This family was initially classified using comparative genomics and is defined by the presence of a characteristic zinc metallopeptidase gluzincin motif (HEXXHX(8,28)E) (20). This superfamily contains sequences that span multiple MEROPS peptidase families (21) but may share the property of O-glycoprotein recognition and cleavage. While some members cleave O-glycosylated non-mucin proteins, others are selective for mucin-domain glycoproteins and are therefore referred to as mucinases (Fig. 1*A*). Thus far, M60-like domain-containing proteases BT4244 from *Bacteroides thetaiotaomicron*, ZmpA, ZmpB, and ZmpC from *Clostridium perfringens*, IMPa from *Pseudomonas aeruginosa*, and Amuc_0627 (AM0627), Amuc_0908 (AM0908), and Amuc_1514 (AM1514) from *A. muciniphila* have been biochemically and/or structurally characterized (22, 23, 24, 25, 26). These analyses revealed specific recognition of O-glycan–containing motifs, resulting in hydrolysis of the peptide backbone of O-glycosylated substrates. With the exception of ZmpA, which was shown to be inactive (23), the enzymes cut N-terminally to O-glycosylated serine or threonine residues.

**Figure 1:**
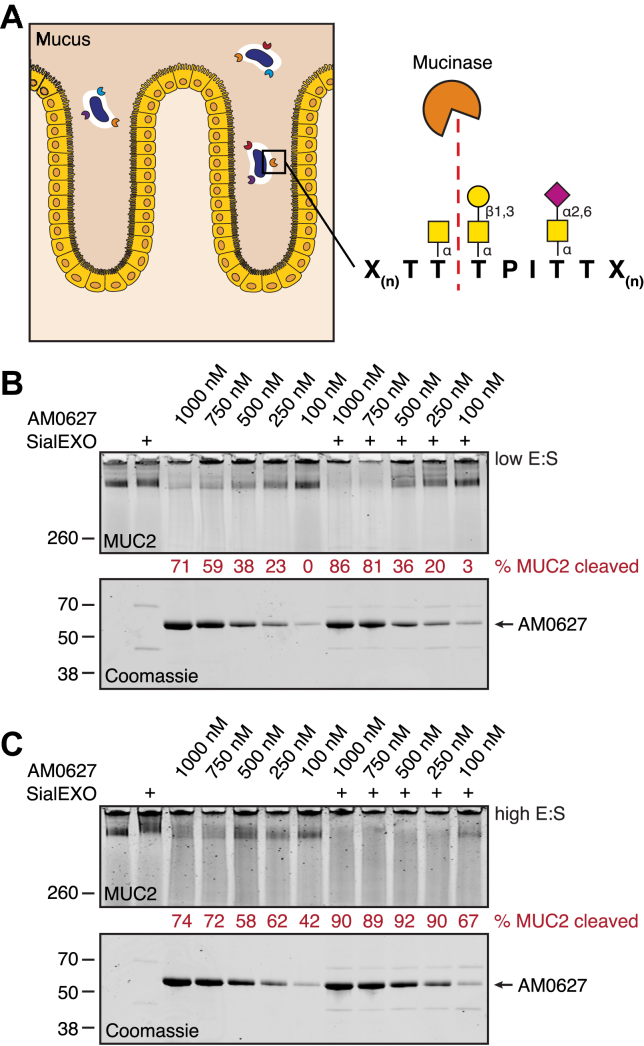
**AM0627 cleaves purified MUC2 *in vitro*.***A*, schematic depicting cleavage of intestinal mucins by secreted mucinases. *B* and *C*, IRdye 800CW-labeled MUC2 derived from LS174T cells was incubated with varying concentrations of AM0627 ± 1 U SialEXO at *low* (*B*) or *high* (*C*) enzyme-to-substrate (E:S) ratio for 22 h at 37 °C. Digests were separated by SDS-PAGE and visualized by in-gel fluorescence. MUC2, mucin 2.

Unique to AM0627 is the minimum requirement for adjacent O-glycans in the cleavage motif (25), which is difficult to rationalize based on primary sequence alone. In addition, this particular enzyme has been shown to be highly upregulated both at the transcript and protein levels *in vitro* when *A. muciniphila* is grown in the presence of porcine gastric mucin (27), indicating that this enzyme may have a prominent role in the breakdown of host glycoproteins. Here, we present the crystal structure of AM0627 at 1.9 Å resolution. We then combine docking and site-directed mutagenesis studies to probe the molecular basis for the enzyme’s substrate specificity. These results provide further insight into the structural determinants of O-glycan recognition and expand our current understanding of mucin processing in the gut by *A. muciniphila*.

## Results

### Purified MUC2 is proteolyzed by AM0627 *in vitro*

The cleavage motif of AM0627 was previously determined by characterizing activity against recombinant mucin-domain glycoproteins leukosialin (CD43), P-selectin glycoprotein ligand 1 (PSGL-1), mucin 16 (MUC16), and podocalyxin using mass spectrometry (25). This enzyme was shown to cleave between two O-glycosylated serine or threonine residues bearing truncated glycans such as the Tn (O-GalNAc) or T (O-GalNAc-galactose) antigens, with a preference for desialylated substrates. Since intestinal mucus layers in the colon are organized around mucin 2 (MUC2) (28), we tested AM0627 for its ability to cleave MUC2 with or without removal of sialic acids. MUC2 was purified from the LS174T colon cancer cell line (29) (Fig. S1), labeled with a fluorophore *via N*-hydroxysuccinimide chemistry, and incubated with varying concentrations of AM0627 overnight at 37 °C. At a low enzyme-to-substrate (E:S) ratio, AM0627 was capable of digesting MUC2 at concentrations above 100 nM. At higher AM0627 concentrations, the activity improved with desialylation of MUC2 using SialEXO, a commercially available mixture of two sialidases from *A. muciniphila* (Figs. 1*B* and S2*A*). The activity was tested again using half the amount of MUC2 to raise the E:S ratio for both AM0627 and SialEXO, increasing the percentage of MUC2 cleaved at all concentrations. At this higher ratio, the degree of desialylation improved, resulting in a larger gel shift of the sialidase-treated MUC2 and allowing maximal activity to be achieved across a wider range of AM0627 concentrations (Figs. 1*C* and S2*B*). These results further highlight the enzyme’s preference for desialylated substrates.

### AM0627 shares structural O-glycan recognition properties of characterized PF13402 enzymes

**Figure 2:**
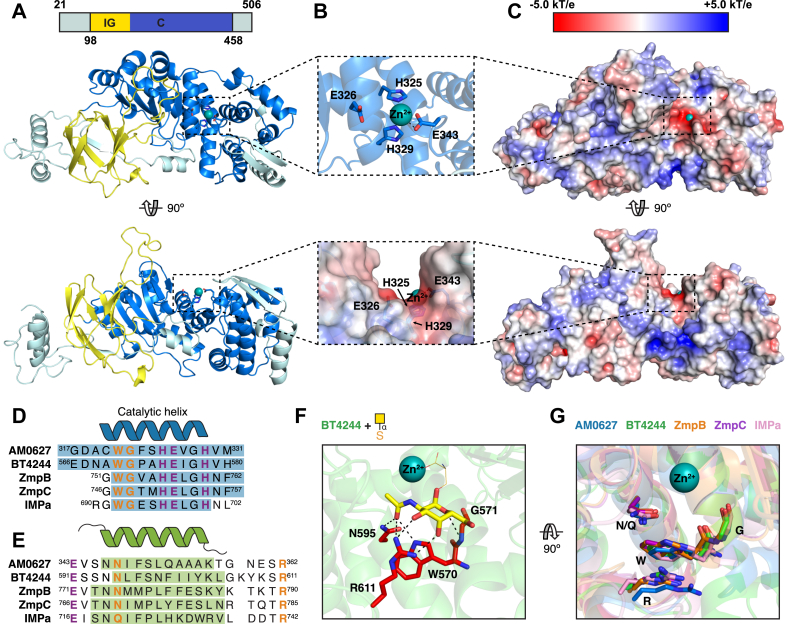
**Structure of AM0627 reveals O-glycan recognition properties shared between PF13402 enzymes.***A*, cartoon representation of AM0627 with the catalytic domain (C) in *blue* and immunoglobulin-like domain (IG) in *yellow*. *B*, cartoon (*upper panel*) and electrostatic surface (*lower panel*) representations of the active site with the Zn^2+^ ion represented as a *teal sphere* and the gluzincin motif residues shown as *sticks*. *C*, electrostatic surface representation of AM0627. *D* and *E*, catalytic helix (*D*) and S1′ subsite helix (*E*) sequence alignments between active, structurally characterized PF13402 enzymes determined using the DALI server (31). Zn^2+^ ion-binding residues are highlighted in *purple* while G1′ GalNAc-binding residues are shown in *orange*. Colored regions highlight helix sequences. *F*, Tn antigen-BT4244 interface (22) depicting conserved G1′ GalNAc-binding residues as *red sticks*, GalNAc as *yellow sticks*, the glycosylated serine as *orange lines*, the Zn^2+^ ion as a *teal sphere*, and hydrogen bonds as *black dashes*. *G*, superposition of AM0627 (*blue*), BT4244 (*green*, PDB ID: 5KD8), ZmpB (*orange*, PDB ID: 5KDU), ZmpC (*purple*, PDB ID: 6XT1), and IMPa (*pink*, PDB ID: 5KDX) active sites showing the conserved G1′ GalNAc-binding subsite residues. The Zn^2+^ ion in AM0627 is shown as a *teal sphere*.

To probe the molecular basis for O-glycan specificity, we determined the structure of AM0627 in its unliganded form, with the signal peptide (residues 1–20) removed, at 1.9 Å resolution *via* X-ray crystallography (Fig. 2, *A*–*C* and Table S1). AM0627 consists of an immunoglobulin-like fold domain followed by a catalytic domain (Fig. 2*A*). The active site harboring catalytic residue E326 contains a beta strand and two alpha helices arranged within the larger gluzincin motif (30), characterized by histidine residues H325 and H329 and glutamate residue E343 bound to a zinc ion (Fig. 2*B*). A search for structural homologs using the DALI server (31) (Table S2) revealed high structural similarity with other PF13402 proteases known to recognize O-glycans (entries 2–6 in Table S2) (Fig. S3). Sequence alignments of AM0627 with active, structurally characterized PF13402 enzymes (Fig. S4) highlighted conserved tryptophan, glycine, asparagine/glutamine, and arginine residues (Fig. 2, *D* and *E*) that interact with the initiating α-GalNAc of mucin-type O-glycans, as exemplified by a cocrystal structure obtained by Noach *et al*. (22) of BT4244 with a serinyl Tn antigen (Fig. 2*F*). Notably, these S1′ subsite residues are spatially conserved between AM0627, BT4244, ZmpB, ZmpC, and IMPa (Fig. 2*G*), providing a rationale for the specificity of these proteases for substrates carrying mucin-type O-glycosylation, a property that may be shared by the entire PF13402 family.

### Molecular modeling provides a structural rationale for recognition of adjacent truncated O-glycans

Beyond the conserved S1′ subsite residues that recognize the initiating α-GalNAc, PF13402 enzymes are known to contain additional, less conserved glycan-binding subsites that result in unique cleavage motifs (22). These subsites were defined by Noach *et al*. (22) as the region surrounding the initiating sugar residue (G1′) as well as residues branching from the O6 (G2′) and O3 (G2’’) positions. We turned to molecular modeling (i) to better understand how AM0627 recognizes glycans extending beyond the initiating GalNAc residue at the P1′ position and (ii) to identify the residues unique to AM0627 that are involved in recognizing glycans branching from the P1 position.

**Figure 3:**
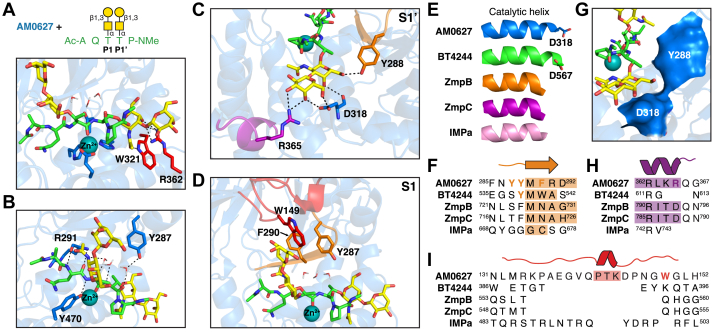
**Molecular modeling reveals residues involved in the recognition of adjacent truncated O-glycans.***A*, docking of glycopeptide Ac-AQT^∗^T^∗^P-*N*Me into AM0627, where *asterisks* indicate glycosylated residues. Gluzincin motif residues are shown as *blue sticks* and S1′ subsite G1′ GalNAc-binding residues are shown as *red sticks*. *B*, glycopeptide backbone contacts with AM0627 and inserted active site water molecules. *C* and *D*, nonconserved and semiconserved S1’ (*C*) and S1 (*D*) subsite glycan-interacting residues. In (*A–D*), the Zn^2+^ ion is represented as a *teal sphere* and hydrogen bonds are depicted as *black dashes*. *E*, comparison of catalytic helices between PF13402 enzymes. The spatially conserved aspartate residues in AM0627 and BT4244 are shown as *sticks*. *F*, active site beta strand sequence alignments between PF13402 enzymes determined using the DALI server (31). *G*, surface representation of residues Y288 and D318 in the S1′ subsite. *H*, G2’’ glycan-binding S1′ subsite sequence alignments between PF13402 enzymes determined using the DALI server. *I*, S1 subsite loop sequence alignments between PF13402 enzymes determined using the DALI server. In (*F*, *H* and *I*), colored regions highlight strand/helix sequences.

The active site beta strand is positioned further from the gluzincin motif residues in AM0627 compared to related enzymes (32), preventing the formation of hydrogen bonds typically observed between metallopeptidases and their ligands (33). Thus, the beta sheet formed by this strand and two additional strands was elongated to facilitate contacts with the ligand. A PSGL-1–based peptide fragment carrying adjacent T antigens was then docked into the active site of AM0627 (Fig. 3, *A*–*D*). In the final enzyme–substrate complex, the position of the residues, including those of the beta sheet, changed very little, reflecting a reasonable induced fit (Fig. S5).

In the S1′ site, the conserved G1′ GalNAc-binding residues allowed docking of the T antigen with good confidence, creating expected contacts with residues W321 and R362 (Fig. 3*A*). Additionally, the semiconserved residues Y288 and D318 hydrogen bond with the G1′ GalNAc (Fig. 3*C*) in a manner similar to what is observed in BT4244 (22). It has previously been suggested that a longer catalytic helix may sterically block the entrance of O-glycopeptides with substitutions at O6 (G2′) (34). Comparison of the catalytic helices between PF13402 enzymes revealed that AM0627 and BT4244 have longer helices containing a spatially conserved aspartate residue (Fig. 3*E*). This residue, together with a semiconserved Y288 (Fig. 3*F*), forms a surface that sterically blocks accommodation of a G2′ sugar residue (Fig. 3*G*). In contrast, the catalytic helices of ZmpB, ZmpC, and IMPa are shorter, and these enzymes do not share the tyrosine residue present in AM0627 and BT4244, enabling hydrolysis of α-2,6-sialylated O-glycopeptides. In the G2’’ subsite, a nonconserved arginine residue, R365, was found to bind to the β-1,3-linked galactose (Fig. 3*H*). In IMPa, where there is no structurally conserved residue at this position, the β-1,3-linked galactose points into solvent and does not interact with the enzyme (22). In ZmpB and ZmpC, the shorter aspartate residues at this site also do not interact with the β-1,3-linked galactose (23). Thus, although the T antigen is accommodated, it may be a less preferred substrate for these enzymes compared to AM0627.

Again, AM0627 is unique in that it also requires glycosylation branching from the P1 position. The structure and sequence of AM0627 was compared with related PF13402 enzymes to identify residues that may comprise the S1 site. Notably, a cluster of aromatic residues, formed by a nonconserved W149 present in a long loop (Fig. 3*I*) as well as residues Y287 and F290 in and around the active site beta strand (Fig. 3*F*), was observed adjacent to the active site. Interactions between a hydrophobic cavity and a T antigen-containing glycopeptide were similarly observed in the O-glycoprotease OgpA from *A. muciniphila* (34). Therefore, we used docking to determine how the T antigen might interact with these residues. We observed that the initiating GalNAc moiety only contacted this cluster *via* its acetyl group and that it interacted indirectly with the backbone of the beta strand (M289), side chain of Y287, and backbone of the substrate through a network of added water molecules (Fig. 3, *B* and *D*). Thus, the G1′ subsite of S1 does not appear to be as defined as that of S1’. The galactose moiety, on the other hand, formed a specific H-π interaction with W149 as well as other nonspecific hydrophobic contacts with W149 and F290. Overall, the docking results provide a structural rationale for the recognition of adjacent truncated O-glycans and further highlight the presence of less conserved glycan-binding subsites that diversify this family of enzymes.

### Mutagenesis studies reveal important S1 subsite residues

**Figure 4:**
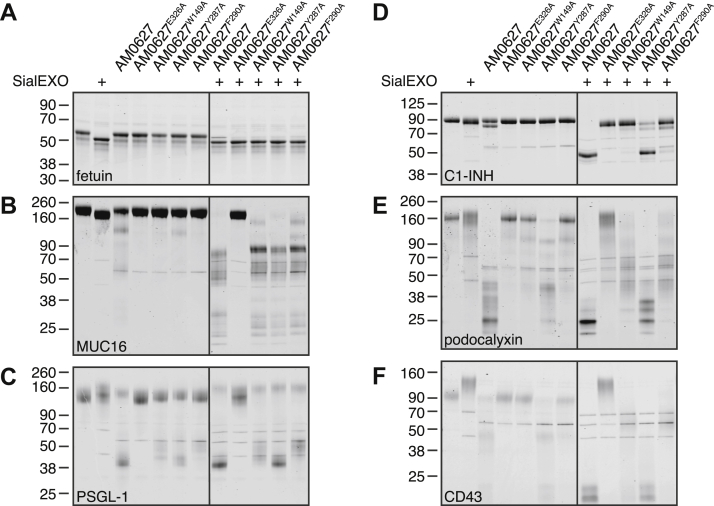
**AM0627 point mutations alter cleavage patterns.***A-F*, fetuin (*A*), recombinant MUC16 (*B*), recombinant PSGL-1 (*C*), purified C1-INH (*D*), recombinant podocalyxin (*E*), and recombinant CD43 (*F*) were incubated with 50 nM AM0627 or AM0627 point mutants ± 1 U SialEXO for 22 h at 37 °C. Digests were separated by SDS-PAGE and visualized using Coomassie stain. Gels were spliced to remove an inactive AM0627 variant that was tested. C1-INH, C1 esterase inhibitor; MUC16, mucin 16; PSGL-1, P-selectin glycoprotein ligand 1.

We performed structure-guided mutagenesis to assess the importance of each S1 subsite residue in glycan recognition. Specifically, residues W149, Y287, and F290 were each mutated to alanine and recombinantly expressed and purified (Fig. S6), with consistent activity, metal dependence, and stability observed between independent preparations (Fig. S7). The *in vitro* activities of each point mutant were then compared to that of WT AM0627. Similarly to AM0627, none of the mutants cleaved fetuin (Fig. 4*A*), which is O-glycosylated but is not a mucin (35). The inability of the point mutants to cleave fetuin, even at a higher E:S ratio (Fig. S8), reflected a maintained selectivity for mucins. We then tested activity against recombinant and purified mucin-domain glycoproteins MUC16, PSGL-1, C1 esterase inhibitor, podocalyxin, and CD43 (Fig. 4, *B*–*F*). A catalytically inactive point mutant, AM0627^E326A^ (25), was included as a control and displayed no activity. As with AM0627, all mutants showed increased activity against desialylated substrates. The cleavage patterns generated by AM0627^Y287A^ were most similar to those of AM0627, whereas AM0627^W149A^ and AM0627^F290A^ typically produced higher molecular-weight products. These differences resulted, at least partially, from altered digestion kinetics (Fig. S9*A*). However, distinct cleavage patterns were also observed with increased enzyme concentrations and incubation times (Fig. S9*B*), indicating that the mutations affected not only kinetics but also substrate recognition properties.

### S1 subsite mutation alters substrate preferences

**Figure 5:**
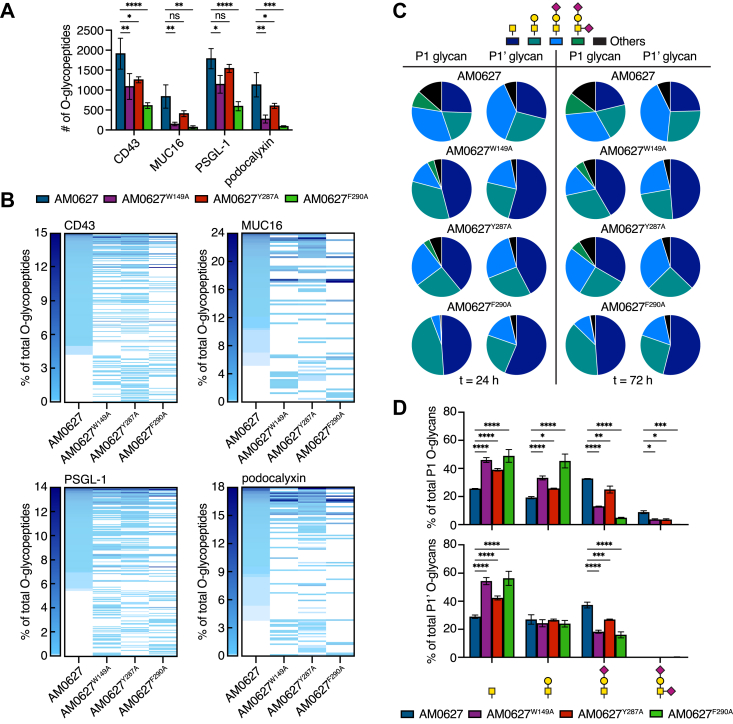
**Mass spectrometry analysis reveals altered substrate preferences.***A*, total number of generated glycopeptides with high confidence O-glycosite localization for each substrate after a 24-h incubation with AM0627 or point mutants. *B*, heat maps depicting the frequency of each O-glycopeptide sequence normalized to the total number of substrate O-glycopeptides generated by the enzyme after 24 h. Peptide sequences are ordered from highest to lowest frequency (top to bottom) for AM0627. Specific sequences and counts are listed in Dataset S3. *C*, distributions of O-glycan occurrences at P1 and P1′ in O-glycopeptides generated by each enzyme after 24- and 72-h digests. *D*, quantification of O-glycan occurrences at P1 (*top*) and P1’ (*bottom*) for 24-h digests. Data are mean ± s.d. (*n* = 2). *p*-values were determined by two-way ANOVA with Dunnett correction. ^∗^*p* < 0.05, ^∗∗^*p* < 0.005, ^∗∗∗^*p* < 0.0005, ^∗∗∗∗^*p* < 0.0001.

To determine how the mutations altered activity, we analyzed the generated peptides by mass spectrometry–based O-glycoproteomics using a combination of collision-based and electron-based fragmentation (36, 37). To achieve maximal cleavage, we used a high E:S ratio of 1:3 with incubation times of 24 and 72 h. Results were filtered to include only glycopeptides with the highest confidence O-glycan localization for all sites (Datasets S1 and S2). In most cases, the mutants generated significantly fewer O-glycopeptides for each substrate relative to AM0627 (Fig. 5*A*). The total number of O-glycopeptides changed very little even with an additional 48-h incubation, indicating that near maximal activity was achieved within 24 h for all enzymes (Fig. S10). The frequencies and occurrences of O-glycopeptide sequences were distinct between mucinases, reflecting altered substrate preferences (Dataset S3). Heat maps were generated to visualize O-glycopeptide frequencies across enzyme treatment conditions, showing a skew toward a low number of high frequency peptides and an abundance of low frequency peptides (Figs. 5*B* and S11). The most frequent peptide sequences found in the AM0627 digests did not always have the highest frequencies in the mutant digests. There were also several unique glycopeptide sequences generated by the mutants that were not produced by AM0627. To analyze the minimum sequence motif for each enzyme, glycopeptides from the 24-h digests were used as input for weblogo.berkeley.edu (38). Additionally, we calculated the percent of O-glycosylated serine and threonine residues at each position within the motif, which describes the ratio of modified residues relative to the total number of serines and threonines at a given position. For peptides identified from all enzymes, greater than 84% and 95% of serine and threonine residues at the P1 and P1′ positions, respectively, were glycosylated, reflecting a retained requirement for adjacent O-glycans with cleavage occurring between the glycosylated residues (Fig. S12). Thus, disruption of the hydrophobic pocket did not hinder the enzyme’s ability to accommodate adjacent glycans in its active site.

To probe changes in O-glycan preferences, the frequencies of each glycan structure at the P1 and P1′ positions were analyzed. For the mutants, a greater proportion of generated O-glycopeptides carried Tn and T antigens at the P1 position relative to AM0627. This skew toward the Tn antigen was also observed at the P1′ position, with larger changes occurring for AM0627^W149A^ and AM0627^F290A^ compared to AM0627^Y287A^. The observed shifts were maintained even with longer incubation times (Figs. 5, *C* and *D* and S13). Thus, altering the structure of one subsite influenced glycan accommodation at the other. These results demonstrated that although disruption of the hydrophobic pocket did not change the requirement for adjacent O-glycans, the mutations did affect the glycan preferences.

## Discussion

The Pfam 13402 family contains peptidases that have been shown to rely on substrate O-glycosylation for activity (22, 23, 25). Of the enzymes characterized to date (32), AM0627 from *A. muciniphila* is unique in its strict requirement for adjacent O-glycosylated residues. In addition, this enzyme is highly upregulated during growth on mucin (27) and likely plays a role in the processing of host glycoproteins as a source of nutrients, a concept that is further supported by its activity against MUC2 (Fig. 1, *B* and *C*). To better understand the molecular determinants driving substrate recognition, we obtained a crystal structure of AM0627 and performed molecular modeling to identify important residues. We then demonstrated that the enzyme’s substrate preferences could be altered through site-directed mutagenesis of these residues.

As with other PF13402 enzymes, AM0627 shared spatially conserved GalNAc-binding residues in its S1′ subsite (Fig. 2*G*) that enable recognition of substrates carrying mucin-type O-glycosylation at P1’. Unique to AM0627 was the presence of a long loop (Fig. 3*I*) that, based on molecular docking, could interact with a glycan at the P1 position *via* formation of a hydrophobic pocket consisting of residues W149, Y287, and F290 (Fig. 3*D*). Although mutation of each of these residues to alanine did not affect the requirement for adjacent glycosylated residues (Fig. S12), they did alter overall activity (Figs. 4 and S10*A*) and substrate preferences (Figs. 5*A* and S10*B*) down to the glycan level (Figs. 5*C* and *D* and S13). In general, the F290A mutation had the largest effect and resulted in the lowest number of generated O-glycopeptides, possibly because this mutation disrupts the hydrophobic pocket and the active site beta sheet involved in recognition of the substrate backbone.

Notably, all three mutations skewed the enzyme’s preference toward the Tn antigen at the P1 and P1′ positions, even though the S1′ subsite was not altered directly (Figs. 5, *C* and *D* and S13). Thus, changing glycan accommodation at one subsite influenced the preferred adjacent glycan, reflecting the likely dynamic nature of this enzyme. Additionally, disrupting the hydrophobic pocket did not prevent accommodation of the T antigen at the P1 position, despite predicted interactions of the galactose moiety with specific residues in the pocket (Fig. 3*D*). This suggests that the enzyme adapts the binding subsites to recognize different pairs of O-glycans, and while the mutations do change the binding dynamics, the specific changes are not easily predictable. Interestingly, the sialyl-T antigen was found at comparable frequencies to the Tn and T antigens at both P1 and P1′, suggesting that the higher activity observed on desialylated substrates is not a result of the enzyme’s inability to accommodate sialylated O-glycans. Instead, sialidase treatment likely induces more complex substrate-specific changes, both at the peptide and glycan levels (39, 40), that facilitate cleavage by AM0627. Cocrystal structures with bound glycopeptide ligands would better elucidate structural changes that occur with the binding of peptides carrying different pairs of O-glycans.

It is also possible that interactions outside of the active site influence glycan preferences within the active site. This phenomenon has been observed for other PF13402 enzymes, where the presence of additional carbohydrate-binding domains and their arrangement in three-dimensional space promotes the accommodation of extended mucins (23, 41). To note, AM0627 is relatively small and does not have any annotated carbohydrate-binding modules, preventing higher-ordered mucin recognition. Instead, the intrinsic requirement for adjacent O-glycosylated residues may allow AM0627 to selectively target densely glycosylated mucins where adjacent O-glycans are more likely to be present.

The structure and molecular modeling presented in this work highlight the diverse nature of glycan-binding subsites within the PF13402 family. Subsite residues involved in glycan recognition are not always easy to predict based on primary amino acid sequence, but conserved and semiconserved glycan-binding subsite residues have been validated through structural studies. A deeper understanding of these subsites will enable the engineering of O-glycoproteases with specific O-glycan preferences to generate novel research tools, similar to what has been done with lectins (42).

Further characterization and engineering of AM0627 could also be relevant in studying the link between the mucin-degrading abilities of *A. muciniphila* and its known health benefits. Supplementation of mucin O-glycans to diet has been shown to promote the outgrowth of *A. muciniphila*, which breaks down mucins to produce short-chain fatty acids (16, 17). Short-chain fatty acids have been linked to improved colonic integrity and are known to have anti-inflammatory functions (43), indicating that the mucin-degrading capabilities of *A. muciniphila* may be involved in preventing dysbiosis. Based on the structure and activity of AM0627, it is likely that this enzyme plays a prominent role in mucin processing. However, further characterization of AM0627 in its native biological context is necessary to fully understand its role in mucin degradation, how it promotes the colonization of niche mucosal environments, and its connection to the observed health benefits of *A. muciniphila*.

## Experimental procedures

### Mucinase cloning, expression, and purification

A Q5 site-directed mutagenesis kit (New England Biolabs) was used to generate mutations in pET28b-AM0627-Δ20-NHis (25) using primers listed in Table S3. BL21(DE3) *E. coli* (New England Biolabs) were transformed with plasmids and grown at 37 °C, 225 rpm in LB (Thermo Fisher Scientific) with 50 μg/ml kanamycin until an optical density of 0.6 to 0.8 was reached. The cultures were induced with 0.4 mM IPTG and grown for an additional 3 to 4 h at 37 °C. The cultures were spun down at 10,000*g* for 10 min at 4 °C. For AM0627, the cell pellet was resuspended in 20 mM Tris–HCl, 500 mM NaCl, pH 8.5, then lysed using a dounce homogenizer followed by sonication with a Fisherbrand Model 705 Sonic Dismembrator (Thermo Fisher Scientific). The lysate was applied to a 5 ml HisTrap HP column (Cytiva Life Sciences) using an ÄKTA Pure FPLC. After washing with 25 column volumes of 20 mM Tris–HCl, 500 mM NaCl, 20 mM imidazole, pH 8.5, elution was performed using a linear gradient to 500 mM imidazole. Fractions containing pure protein as judged by SDS-PAGE analysis were concentrated using Amicon Ultra 10 kDa MWCO filters (Millipore Sigma), dialyzed into PBS, pH 6.5 using 10K MWCO Slide-A-Lyzer Dialysis Cassettes (Thermo Fisher Scientific), and stored at −80 °C.

For AM0627 mutants, cell pellets were lysed in xTractor buffer (Clontech), and lysates were applied to 0.5 ml of Ni-NTA resin (Qiagen). After washing with eight column volumes of 20 mM Tris–HCl, 500 mM NaCl, 20 mM imidazole, pH 8.5, proteins were eluted with 6 × 250 μl of wash buffer supplemented with 250 mM imidazole. Fractions containing pure protein as judged by SDS-PAGE analysis were dialyzed into PBS, pH 6.5 using 10K MWCO Slide-A-Lyzer Dialysis Cassettes and stored at −80 °C.

### MUC2 purification and Western blot

The MUC2 purification protocol was adapted from (44). Briefly, LS174T cells (ATCC) were maintained at 37 °C, 5% CO_2_ in dulbecco’s modified eagle’s medium (DMEM) supplemented with 10% fetal bovine serum and 1% penicillin/streptomycin. At confluency, cells were washed once with 5 ml of PBS, resuspended in at least 5× the volume of cold 6 M guanidine hydrochloride (GuHCl), 5 mM EDTA, 10 mM NaH_2_PO_4_, pH 6.5, and solubilized by rotating at 4 °C for 24 h. Insoluble mucins were precipitated by centrifugation at 18,000 rpm for 30 min at 4 °C. The wash step was repeated five times in the same GuHCl buffer with incubation times of at least 3 h. The pellet was solubilized in 6 M GuHCl, 100 mM Tris, 5 mM EDTA, 25 mM DTT, pH 8.0 for 5 h at 37 °C. Iodoacetamide was added to 62.5 mM and the solution was rotated overnight at room temperature in the dark. The sample was spun down at 10,000 rpm for 30 min at 4 °C to remove insoluble material, supernatants were dialyzed against water for 36 h using 10K MWCO Slide-A-Lyzer Dialysis Cassettes, and aliquots were stored at −80 °C. To validate enrichment of MUC2, 9 μl of each wash and the purified product were loaded onto a 3 to 8% Criterion XT Tris-Acetate protein gel (Bio-Rad) and run in XT-Tricine (Bio-Rad) at 150 V. Total protein was quantified using a SilverXpress Silver Staining Kit (Thermo Fisher Scientific). For Western blot, 15 μl of each wash and the purified product were loaded onto a 3 to 8% Criterion XT Tris-Acetate protein gel (Bio-Rad) and run in XT-Tricine (Bio-Rad) at 150 V. The gel was transferred to a 0.2 μm nitrocellulose membrane using the Trans-Blot Turbo Transfer System (Bio-Rad) at 2.5 A for 15 min. MUC2 staining was performed using an anti-MUC2 antibody (996/1) (Abcam) and IRDye 800CW Goat anti-Mouse IgG (LI-COR Biosciences) according to manufacturer recommendations.

### MUC2 labeling

IRDye 800CW NHS Ester (LI-COR Biosciences) was dissolved at 10 mM in dimethyl sulfoxide. IRDye 800CW NHS Ester (1 mM) was incubated with 2 mg of MUC2 (measured *via* lyophilization) in PBS supplemented with 100 mM K_2_HPO_4_ for 3 h at room temperature, rotating end-over-end in the dark. Free dye was removed *via* dialysis against water using a 100KD Spectra/Por Float-A-Lyzer G2 Dialysis Device (Thermo Fisher Scientific) and aliquots were stored at −20 °C.

### *In vitro* digests

Digests against IRDye 800CW-MUC2 (2.25 μg or 4.5 μg) were performed with varying concentrations of AM0627 ± 1 U of SialEXO (Genovis) in PBS (12 μl of total volume) for 22 h at 37 °C. Reactions were loaded onto a 4 to 12% Criterion XT Bis-Tris protein gel (Bio-Rad) and run in XT-MOPS (Bio-Rad) at 180 V. Gels were imaged using an Odyssey CLx Near-Infrared Fluorescence Imaging System (LI-COR Biosciences).

Recombinantly expressed podocalyxin, MUC16, PSGL-1, and CD43 were purchased from R&D Systems (1658-PD, 5609-MU, 3345-PS, and 9680-CD, respectively). C1 esterase inhibitor purified from human plasma was purchased from Innovative Research (IHUC1INH1MG). Fetuin was purchased from Promega (V4961). Digests against recombinant and purified substrates (1 μg) were performed with 50 nM AM0627 or AM0627 mutants ± 1 U of SialEXO in PBS (9 μl of total volume) for 22 h at 37 °C. Reactions were loaded onto a 4 to 12% Criterion XT Bis-Tris protein gel and run in XT-MOPS at 180 V. Protein was visualized with Acquastain Protein Gel Stain (Bulldog-Bio).

### Crystallization, data collection, and structure determination

AM0627 was concentrated to 10.5 mg/ml in 100 mM Tris, 150 mM NaCl, 1 mM ZnCl_2_, pH 8.0. Crystals were obtained using sitting-drop vapor diffusion for screening and sitting- or hanging-drop vapor diffusion for optimization at 6 °C or 16 °C. Crystallization experiments were set up using a Douglas Oryx8 Nanodrop dispensing robot (Douglas Instruments Ltd). Initial diffraction-quality AM0627 crystals were obtained in 30% polyethylene glycol 500 monomethyl ether, 10% polyethylene glycol 20,000, in a buffer at pH 6.5. Crystals were harvested and cryocooled by plunging in liquid nitrogen with or without addition of cryoprotectant. Data to Bragg spacings of 2.5 Å and 2.8 Å were collected at 100 K on beamlines BL14-1 and BL12-2 (45) at the Stanford Synchrotron Radiation Lightsource (SSRL) at SLAC National Accelerator Laboratory. Potential model structures for molecular replacement were identified using a sequence-structure homology search with FUGUE (46). The two structures above the certainty threshold score were an M60-like protease from *B. thetaiotaomicron* VPI-5482 (PDB ID: 5KD5 (22)) and a functionally uncharacterized protein from *Bacillus anthracis* (PDB ID: 4FCA). The search failed to provide a solution to these initial crystals in space group P2_1_ (two different unit cells containing four and eight copies of the polypeptide in the asymmetric unit, respectively). Probing was extended to archetype MA clan metallopeptidases (47).

While optimizing conditions, new crystals were obtained in 10% polyethylene glycol 4,000, 20% glycerol, 0.1 M HEPES/MOPS, pH 7.5 buffer and a mixture of alcohols (0.02 M each of 1,6-hexanediol, 1-butanol, 1,2-propanediol, 2-propanol, 1,4-butanediol, and 1,3-propanediol) by sitting-drop vapor diffusion. Data to a Bragg spacing of 2.65 Å were collected at the SSRL beamline BL9-2 showing a hexagonal setting with one copy of the polypeptide in the asymmetric unit. This rendered the phase problem tractable and proved to be crucial for successful determination of the structure. A combination of automated molecular replacement and manual adjustment of partially built models was employed. MrBUMP (48) traced an incomplete model from a morphed 5KD5 structure with CHAINSAW (49), Phaser.sculptor (50), using PARROT/BUCCANEER (51) for density modification and automated model building, and PHASER or MOLREP (52) for molecular replacement. This partial model, initially consisting of residues 300 to 500, was completed by successive rounds of density modification and automated building cycled with manual adjustment and model building with COOT (53) and refinement with REFMAC (54). Except for a short segment at the N-terminus, residues 58 to 506 were traced in the electron density maps. This partial model was then used to phase a 1.9 Å dataset collected at SSRL beamline BL12-2 from a crystal grown from optimized crystallization conditions with additives. This allowed unambiguous tracing of residues 20 to 506 in the structure. Refinement progressed to convergence and reached an excellent agreement to the experimental data (Table S1). The largest electron density peak evident after structure solution accounted for the single, tetrahedrally-coordinated Zn^2+^ ion in the polypeptide chain. Further extra density near the zinc atom was interpreted as a formate molecule (presumably from additive screening), which coordinates the zinc as a monodentate ligand. Additional formate molecules, a chlorine atom, and a polyethylene glycol molecule were added in later stages of refinement. Solvent water molecules were first assigned based on their hydrogen bonding properties; later, additional water molecules were automatically added. Data were reduced with XDS (55), scaled with SCALA (56), and analyzed with different software routines within the CCP4 suite (57). Molecular graphics were generated using PyMOL (58).

### Molecular modeling

All molecular modeling was performed using Molecular Operating Environment (MOE 2020.09) (59). Briefly, a generic peptide substrate was constructed in the active site of AM0627, and glycans were sequentially grafted onto residues in the P1′ and P1 positions. The sequence of the resulting species was modified to yield a PSGL-1-based peptide fragment, which was docked into the active site.

As before (60, 61), the crystal structure of AM0627 was prepared by (i) removing all crystallized solvent molecules and (ii) subsequently adding unresolved side chains and hydrogens so that all atoms obtained proper valency and charge. The resulting structure was then overlaid with X-ray crystal structures of ZmpB (PDB ID: 5KDN) (22), OgpA (PDB ID: 6Z2D) (34), astacin (PDB ID: 1QJI) (62), and serralysin (PDB ID: 3VI1), and modeled structures of StcE (60) and CpaA (61) using the conserved histidine residues and zinc ions of their active sites. A generic peptide (Ac-AATTA-*N*Me) was constructed based on the peptide portions of ligands cocrystallized with OgpA, astacin, and serralysin as well as those previously modeled into StcE and CpaA. The T antigen portion of the ligand cocrystallized with ZmpB was grafted onto the second threonine residue present in the P1′ position. The side chain of threonine typically adopts *gauche*^*+*^ and *trans* conformations (63); here, the threonine was modeled in the *trans* conformation similar to the crystal structure. The conserved (W321, R362), semiconserved (Y288), and nonconserved (R365) residues of the S1′ site were sequentially minimized in the Amber10:EHT force field (64) to ensure they formed proper contacts with the newly constructed glycan; all other atoms were held fixed during each minimization. The β-sheet formed by the active site beta strand and two adjacent strands (V279→G300) was lengthened to facilitate enzyme-ligand contacts. Restraints were used to ensure that all hydrogen bonds within the sheet and to the residue in the P2 position were maintained (between 1.7 and 2.5 Å). Finally, both the residues of the β-sheet and the substrate were allowed to minimize in the Amber10:EHT force field. All other residues were held fixed during the entire process.

The same crystallographic T antigen was grafted onto the first threonine residue present in the P1 position in the *gauche*^*+*^ conformation, as this positioned the glycan adjacent to a cluster of aromatic residues. The three residues of this presumed S1 site were minimized together in the Amber10:EHT force field; all other atoms were held fixed. The sequence of the generic peptide was then modified to reflect a PSGL-1–based fragment (Ac-AQT^∗^T^∗^P-*N*Me), with side chains being added in their lowest-energy conformation. Three water molecules were added to the active site to mediate hydrogen bonding between the enzyme and substrate. Finally, the peptide substrate as well as the zinc ion, solvent, and enzyme residues within 9 Å of the substrate were minimized together in the Amber10:EHT force field; as before, all other atoms were held fixed during this step.

### MS sample preparation

Digests against recombinant glycoproteins podocalyxin, MUC16, PSGL-1, and CD43 (3 μg) were performed with AM0627 or AM0627 mutants at a 1:3 E:S ratio in PBS (20 μl of total volume) for 24 or 72 h at 37 °C. Control digests were set up with substrate alone. Digests were de–N-glycosylated *via* addition of 1 μl of PNGase F (New England Biolabs, diluted to 50,000 U/ml in PBS) for 8 h at 37 °C. DTT was added to a final concentration of 10 mM and samples were incubated at 55 °C for 15 min. Samples were brought to room temperature, then chloroacetamide was added to a final concentration of 40 mM and incubated in the dark at room temperature for 30 min. Trypsin (Promega) was added at a 1:15 E:S ratio and samples were incubated overnight at room temperature. Reactions were quenched by dilution with 500 μl of 0.2% formic acid (FA) in water and peptides were desalted using 10 mg/1 ml Strata-X columns (Phenomenex). Briefly, columns were wet with 1 ml of acetonitrile followed by 1 ml of 0.2% FA. Acidified peptides were loaded onto the columns and washed with 300 μl of 0.2% FA. Peptides were eluted with 400 μl of 0.2% FA, 80% acetonitrile, dried *via* lyophilization, then resuspended in 10 μl of 0.2% FA.

### LC-MS/MS

Data was acquired using product-dependent triggering of EThcD scans as previously described (36). Approximately 1 μg of peptides were injected on the column for each sample. Peptides were separated over a 25 cm Aurora Series UHPLC reversed phase LC emitter column (75 μm inner diameter packed with 1.6 μm, 160 Å, C18 particles, IonOpticks). A Dionex Ultimate 3000 RPLC nano system (Thermo Fisher Scientific) with an integrated loading pump was used for online liquid chromatography using mobile phases A (0.2% FA in water) and B (0.2% FA in acetonitrile). Peptides were loaded onto a trap column (Acclaim PepMap 100 C18, 5 μm particles, 20 mm length, Thermo Fisher Scientific) at 5 μl/min, which was put in line with the analytical column 5.5 min into the acquisition. Gradient elution was performed at 300 nl/min. The gradient was held at 0% B for the first 6 min of the analysis, followed by an increase from 0% to 5% B from 6 to 6.5 min, an increase from 5% to 22% B from 6.5 to 156.5 min, an increase from 22% to 90% B from 156.5 to 160 min, isocratic flow at 90% B from 160 to 164 min, and a re-equilibration at 0% for 16 min for a total analysis time of 180 min. Eluted peptides were analyzed on an Orbitrap Fusion Tribrid MS system (Thermo Fisher Scientific). Precursors were ionized using an EASY-Spray ionization source (Thermo Fisher Scientific) held at +2.2 kV compared to ground, and the inlet capillary temperature was held at 275 °C. Survey scans of peptide precursors were collected in the Orbitrap from 400 to 1800 m/z with a normalized AGC target of 100% (400,000 charges), a maximum injection time of 50 ms, and a resolution of 60,000 at 200 m/z. Monoisotopic precursor selection was enabled for peptide isotopic distributions, precursors of z = 2 to 8 were selected for data-dependent MS/MS scans for 3 s of cycle time, and dynamic exclusion was enabled with a repeat count of 2, repeat duration of 20 s, and exclusion duration of 20 s. Priority filters were set to favor highest precursor charge states and lowest precursor m/z values. An isolation window of 2 m/z was used to select precursor ions with the quadrupole. EThcD scans were collected in product-dependent fashion (65), where the presence of oxonium ions (126.055, 138.0549, 144.0655, 168.0654, 186.076, 204.0865, 274.0921, 292.1027, and 366.1395) in a “scouting” higher-energy collisional dissociation (HCD) MS/MS scan triggered acquisition of a second MS/MS scan. The “scout HCD” scan used an automated scan range determination and a first mass of 100 Th, a normalized collision energy of 36, a normalized AGC target value of 100% (50,000 charges), a maximum injection time setting of Auto (54 ms), and a resolution of 30,000 at 200 m/z. If at least four of the nine listed oxonium ions were present in the scout HCD scan within a ±15 ppm tolerance and were among the 20 most intense peaks, an EThcD MS/MS scan was triggered that used calibrated charge dependent parameters for calculating reagent AGC targets and ion-ion reaction times (66), a supplemental collision energy of 25, a scan range of 200 to 4000 m/z, a maximum injection time of 400 ms, a normalized AGC target of 200% (100,000 charges), and a resolution of 60,000 at 200 m/z.

### MS data analysis

All raw data were searched using O-Pair Search implemented in MetaMorpheus (0.0.317), which is available at https://github.com/smith-chem-wisc/MetaMorpheus (37). Files for digestions of each protein from a given digestion condition (*e.g.*, all four proteins digested with AM0627) were searched together in batches with a fasta file containing Uniprot-derived sequences from all four mucins. The “Glyco Search” option was selected, where the O-glycopeptide search feature was enabled with an O-glycan database of 22 glycans (Table S4). The “Keep top N candidates” feature was set to 50, and Data Type was set as HCD with Child Scan Dissociation set as EThcD. The “Maximum OGlycan Allowed” setting was set to 4, where this number represents both the maximum number of O-glycan modifications that could occur on a glycopeptide candidate and the number of times each O-glycan could occur per peptide. Under Search Parameters, both “Use Provided Precursor” and “Deconvolute Precursors” were checked. Peak trimming was not enabled and Top N peaks and minimum ratio were set to 1000 and 0.01, respectively. In Silico Digestion Parameters were set to generate decoy proteins using reversed sequences, and the initiator methionine feature was set to “Variable”. The maximum modification isoforms allowed was 1024, and nonspecific digestion was enabled for peptides ranging from 5 to 60 residues. Precursor and product mass tolerances were 10 and 20 ppm, respectively, and the minimum score allowed was 3. Modifications were set as Carbamidomethyl on C as fixed, and Oxidation on M and Deamidation on N as variable. Note, O-Pair Search returns a single identification representing two spectra, both an HCD and EThcD spectrum. Identifications are made using the HCD spectrum, and the associated EThcD spectrum is used to localize O-glycosites.

The oglyco.psmtsv results file was used for all data processing. Identifications were filtered to include only target matches (T) and identifications with a q-value < 0.01. O-glycopeptide identifications were further filtered to include only Level 1 identifications, which include only identifications with confident and unambiguous O-glycosite localization, and to exclude O-glycopeptides that contained an N-glycosylation sequon (N-X-S/T). Peptide windows for motif generation were mapped from this filtered O-glycopeptide list onto fasta sequences. The percent of O-glycosylated serine and threonine residues was determined by counting the number of modified residues at a given position relative to the total number of serine and threonine residues. Serine and threonine counts were summed, so this is an aggregate value for both residues.

## Data availability

The atomic coordinates and structure factors (code 7SCI) have been deposited in the Protein Data Bank (https://www.wwpdb.org/). The mass spectrometry glycoproteomics data have been deposited to the ProteomeXchange Consortium *via* the PRIDE partner repository (67) with the dataset identifier PXD032164. All other data are contained in this article.

## Supporting information

This article contains supporting information Tables S1–S4, Fig. S1–S13, and Datasets S1–S3.

## Conflict of interest

C. R. B. is a cofounder and scientific advisory board member of Lycia Therapeutics, Palleon Pharmaceuticals, Enable Bioscience, Redwood Biosciences (a subsidiary of Catalent), OliLux Bio, Grace Science LLC, and InterVenn Biosciences.
