# Supplementary material for: Structure-guided mutagenesis of a mucin-selective metalloprotease from Akkermansia muciniphila alters substrate preferences
Source: J Biol Chem. 2022 Apr 9;298(5):101917. doi: 10.1016/j.jbc.2022.101917 (PMC9118916; doi:10.1016/j.jbc.2022.101917)
Supplement: supporting information [file mmc1.pdf]

Supporting Information

Structure-guided mutagenesis of a mucin-selective metalloprotease from *Akkermansia muciniphila* alters substrate preferences

D. Judy Shon, Daniel Fernandez, Nicholas M. Riley, Michael J. Ferracane, Carolyn R. Bertozzi

This file contains Tables S1 to S4 and Figures S1 to S13

Other supplemental materials for this manuscript include the following:

Dataset S1. O-glycopeptides from 24-h digests
Dataset S2. O-glycopeptides from 72-h digests
Dataset S3. O-glycopeptide sequence frequencies

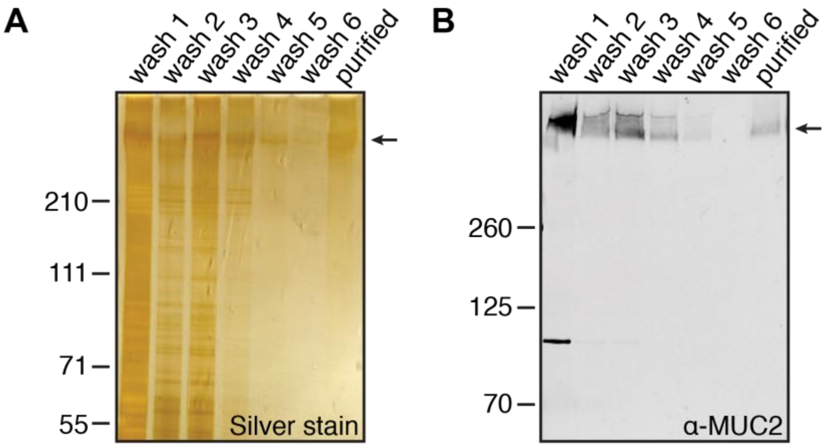

Figure S1. Purification of MUC2. *A*, SDS-PAGE analysis of washes and the purified product quantified using a SilverXpress Silver Staining Kit. *B*, western blot of washes and the purified product with MUC2 antibody (clone 996/1). The MUC2 band is indicated by the black arrow.

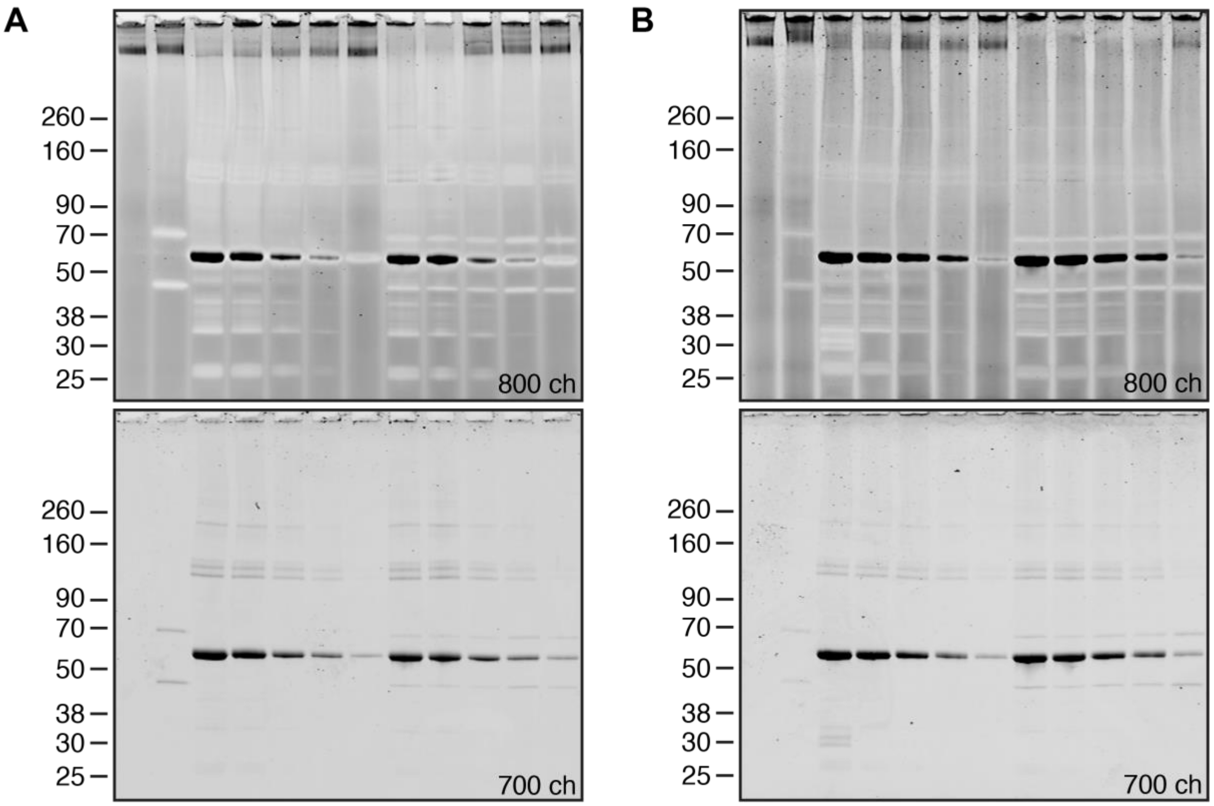

Figure S2. Uncropped MUC2 digest gels. *A* and *B*, uncropped gels corresponding to Fig. 1B-C of IRdye 800CW-labeled MUC2 digests with varying concentrations of AM0627 ± 1 U SialEXO at low (*A*) or high (*B*) enzyme-to-substrate (E:S) ratio for 22 h at 37 ºC.

Table S1. Data collection and refinement statistics

**Data collection**	
Beamline	SSRL BL12-2
Wavelength (Å)	0.97946
Space group	$P6_5$
Cell dimensions	
a, *b*, *c* (Å)	101.56, 101.56, 114.63
α, β, γ (°)	90.00, 90.00, 120.0
Solvent content (%)[a]	60.0
Resolution (Å)[b]	38.21(1.90)
No. of reflections / unique	448,086 / 52,780
R_{merge}[c]	0.105(0.738)
I / σI ratio and $CC_{1/2}$[d]	8.9(1.6) / 0.995(0.681)
Completeness (%)[e]	99.9(99.8)
Redundancy[f]	4.9(3.9)
**Refinement**	
Resolution (Å)	30.0-1.90
No. of reflections / test set	50,020 / 2,666
R_{work} / R_{free}[g]	16.2 / 19.5
F_{obs}-F_{calc} correlation[h]	0.97
No. of atoms	
Protein	3,895
Ligand/ion	1 (zinc) / 1 (chlorine) / 10 (PEG) / 24 (formate)
Water	347
B-factors	
Protein	28.6
Ligand/ion	30.1 (zinc) / 45.0 (chlorine) / 58.1 (PEG) / 48.2 (formate)
Water	38.1
R.m.s. deviations	
Bond lengths (Å)	0.015
Bond angles (°)	1.711
Ramachandran statistics[i]	
Most favored regions (%)	99.5 (415 out of 417 non-proline/non-glycine residues)
Disallowed regions (%)	0.5

[a]Ratio of the volume of the asymmetric unit to the molecular weight of all protein molecules in the asymmetric unit

[b]Value in parentheses is for the highest resolution shell: 1.90 – 2.00 Å

[c]Reliability factor for symmetry-related reflections calculated as: $R_{merge} = \Sigma_{hkl} \Sigma_{j=1 \text{ to } N} | I_{hkl} - I_{hkl}(j) | / \Sigma_{hkl} \Sigma_{j=1 \text{ to } N} I_{hkl}(j)$, where N is the redundancy of the data. In parentheses, the cumulative value at the highest resolution shell

[d]Ratio of mean intensity to the mean standard deviation of the intensity over the entire resolution range and correlation coefficient for random half-datasets for merged data

[e]Fraction of measured reflections to possible observations at the resolution range

[f]Number of measurements of individual, symmetry unique reflections

[g]Average deviation between the observed and calculated structure factors calculated as: $R_{work} = \Sigma_{hkl} ||F_{obs}| - |F_{calc}|| / \Sigma_{hkl} |F_{obs}|$, where the F_{obs} and F_{calc} are the observed and calculated structure factor amplitudes of reflection hkl. R_{free} is equal to R_{factor} but for a randomly selected 5.0% subset of the total reflections that were held aside throughout refinement for cross-validation

[h]Correlation coefficient between observed and calculated structure factor amplitudes

[i]According to Procheck for non-proline and non-glycine residues

Table S2. DALI statistics for top 10 unique hits

PDB	Description	Z-score	RMSD[a] (Å)	No. aligned residues
4FCA	Conserved protein from *Bacillus anthracis* str. Ames	37.9	2.7	374
5KD5	BT_4244 metallopeptidase from *Bacteroides thetaiotaomicron*	34.3	2.6	413
6XSZ	M60 catalytic domain from *Clostridium perfringens* ZmpC	31.9	2.8	374
5KDJ	ZmpB metallopeptidase from *Clostridium perfringens*	26.8	2.9	373
6XSX	Catalytic module of the metalloprotease ZmpA from Clostridium perfringens	25.8	3.0	367
7JTV	IMPa from *Pseudomonas aeruginosa*	22.1	3.2	356
3QNF	Human endoplasmic reticulum aminopeptidase 1 ERAP1	10.5	10.8	233
1Z5H	Tricorn interacting Factor F3 from *Thermoplasma acidophilum*	10.5	8.0	236
4F5C	Pig aminopeptidase N ectodomain	10.4	12.1	235
6U7E	Human aminopeptidase N	10.4	10.3	243

[a]Root-mean-square deviation

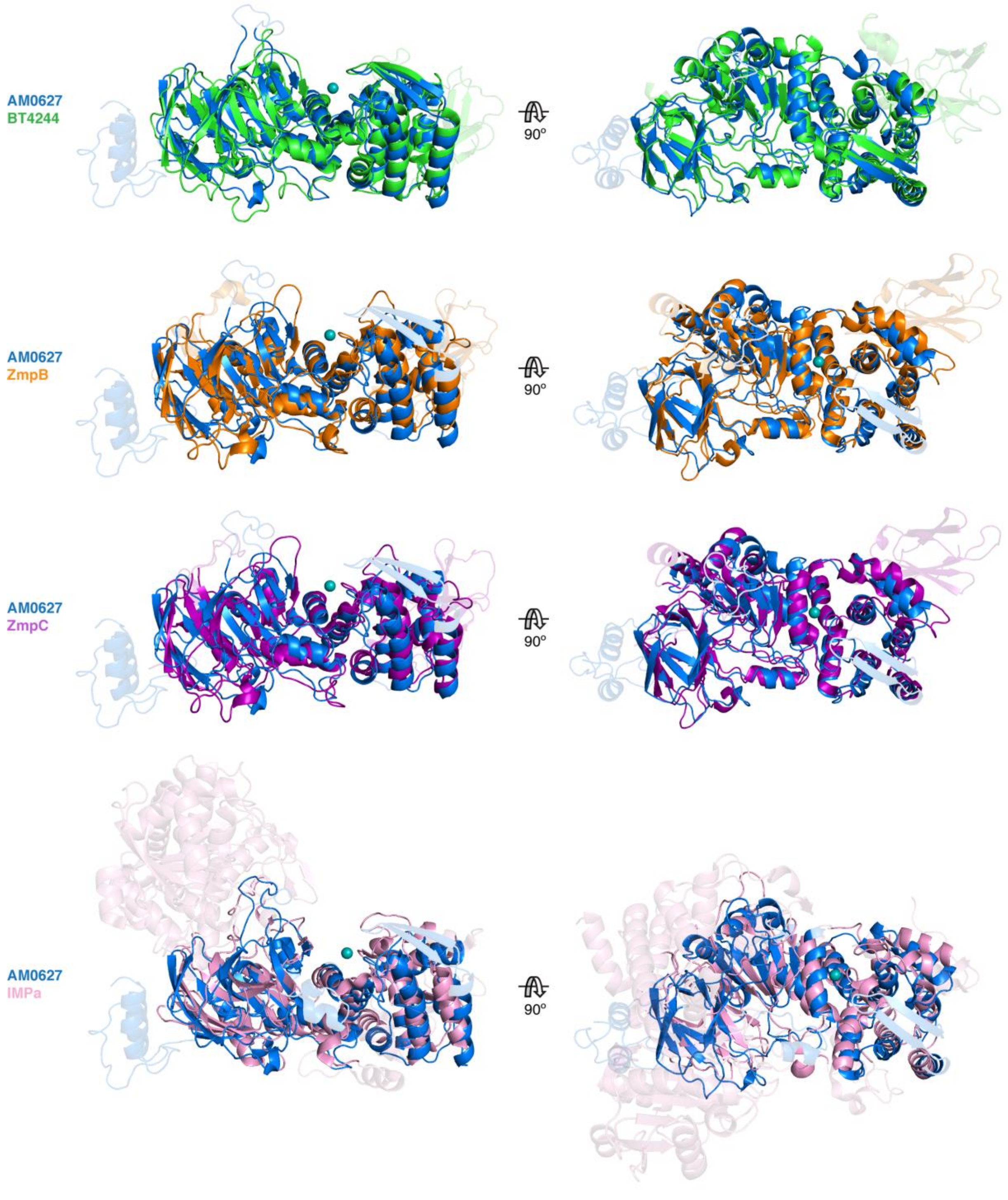

Figure S3. Superposition of PF13402 proteases. Superposition of PF13402 proteases with AM0627. Structurally aligned regions identified using the DALI server are highlighted.

```
AM0627  21 ANTPEHIGNDLKLFKDSSCTSLKPDVKNTSAFQSDAMKELATKILAGHYKPDYLYAEYRALPSPRQTGKNLRIGDGFSKYDNMTGVYLEK-GRH 113
BT4244 325                                                 EKEFRIRSYEPYSNIAEWADKLMTKK-YSDLDNPTGISVKAgDDI 368

       114 VVLVGKTEGQEISLLLPNLMRKPAEGVQPTKDPNGWGLH--KKQIPLKEGINIIDVETPANAYISYFTEDAGK--APKIPVHFV--TGKANGYF 201
       369 IVLVGDTYGQNISMQCIW-ETGT----------EYKQTAssGDVYMLNPGVNKLTMKGEGQLFVMYNT-ELTSntAKPIKIHIPlgSGTVNGFF 450

       202 DTTRGDTNKDWVRLLDQAVSPIMDARGKYIQVAYPVEFLKKFTKDRGTELINAYDKLIGIQYQLMGLDKYGKI---PENRVLARVNFNYYMFRD 292
       451 DLKEHKTDEKYAELLKKSTHKYFCIRGEKIMFYFHRNKLLEYVPNNILSAIHLWDNIVGWQQELMGI--DDVRpsqVNNHLFAISPEGSYMWAS 542

       293 GDGVAYLGNDGTMRMVTDPENVL-KGDACWGFSHEVGHVMQMRPMTWGGMTEVSNNIFSLQAAAKTG-NESRLKRQGSYDKARKEIIEGEIAYL 384
       543 DYQIGFVYT--YLGNILLEDNVMaAEDNAWGPAHEIGHVHQAA-INWASSTESSNNLFSNFIIYKLGkYKSRG---NGLGSVATARYANGQAWY 630

       385 QSK-------DVFNKLVPLWQLHLYFT-KNGHPDFYPDVMEYLRNNAGNYGGNDTVKYQFEFVKACCDVTKTDLTDFFEKWGFFKPGKFHIGDY 470
       631 NMGdathqneDTETHMRMNWQLWIYYHrCEYKTDFWQTLFKLMRE-VNMTEGEDPGKKQLEFAKMASKAANQNLTDFFEMWGFFEPVNTTIEQY 723

       471 AQYDFNVTPEMVEETKKWIAGKGYPKPETDITELSE 506
       724 GTYKYYVSDAMIREAKEYMAQ--FPAPKHAFQYIED 757


AM0627  21 ANTPEHIGNDLKLFKDSSCTSLKPDVKNTSAFQSDAMKELATKILAGHYKPDYLYAEYRALPSPRQTGKNLRIGDGFSKYDNMTGVYLEK-GRH 113
  ZmpB 497                                                      VLELEMRGDSISEAKKRKVW-NFQD-WQITGLSARAgDKI 534

       114 VVLVGKT-EGQEISLLLPNLMRKPAEGVQPTKDPNGWGLHKKQIPLKEGINIIDVET--------------PANAYISYFTEDAGKA-PKIPVH 191
       535 TVYVDVAeGDPTPTLLYKQSLT--------------QHGGATSFQLKPGKNEITIPEinyesngipkdviqGGDLFFTNYK--SDSQkRAPKVR 612

       192 FVTGKANGYFDTTRGDTNKDWVRLLDQA----------VSPIMDARGKYIQVAYPVEFLKKFTK---DRGTELINAYDKLIGIQYQLMGLDKY- 271
       613 IEGASKYPVFILGK-SDENEVMKELEAYvekikaepktTPNIFAVSSNKSLEFVQATYALDWYKknnKTPKYTAEQWDQYIADAMGFWGFDNSk 705

       272 --GKIPENRVLARVNFNY---YMFRDGDGVAYLGNDGTMRMVTDPENVLKGDACWGFSHEVGHVMQMRPMTWGGMTEVSNNIFSLQAAAKTGNE 360
       706 dvNSDFNFRIMPMVKNLSggaFMNAGNGVIGIRPG--NQDAIL---AANK---GWGVAHELGHNFDTGGRT---IVEVTNNMMPLFFESKYKTK 788

       361 SRLKRQGSYDKARKEIIEG----EIAYLQS--KDVFNKLVPLWQLHLYFTKNGHPDFYPDVMEYLRNNAGNYGGNDTVKYQFEFVKACCDVTKT 448
       789 TRITDQNIWENNTYPKVGLddysNNELYNKadSTHLAQLAPLWQLYLYDN-----TFYGKFERQFRERDFGNK--NREDIYKSWVVAASDAMEL 875

       449 DLTDFFEKWGFFKPGKFHIGDYAQYDFNVTPEMVEETKKWIAGKGYPKPETDITELSE 506
       876 DLTEFFARHGIR--------------------vDDKVKEDLA--KYPKPDKKIYYLND 911


AM0627  21 ANTPEHIGNDLKLFKDSSCTSLKPDVKNTSAFQSDAMKELATKILAGHYKPDYLYAEYRALPSPRQTGKNLRIGDGFSKYDNMTGVYLEK-GRH 113
  ZmpC 492                                                      VIELEMRGNSVQEANYRKMWG-FQDW-QVTGLSALAgDKI 529

       114 VVLVGKT-EGQEISLLLPNLMRKPAEGVQPTKDPNGWGLHKKQIPLKEGINIIDVE--------------TPANAYISYFTEDAGKA-PKIPVH 191
       530 TVYVDVEeGEPTPTLLYRQTMT--------------QHGGAKTFQLQNGKNEIVIPeldktsngisegtiQGGELFFTNYN--SDSQtRAPKIR 607

       192 FVTGKANGYFDTTRGDTNKDWVRLLDQA----------VSPIMDARGKYIQVAYPVEFLKKFTK---DRGTELINAYDKLIGIQYQLMGLDKYG 272
       608 IEGAKEYPVFVLGE-SDEDKVIKELEAYvekiekepetTPDIFAVSSNKSLSLTQATYALEWYKnnnKTPKYTAESWDKIVENAMDFWGYDNSS 700

       273 ---KIPENRVLARVNFNY---YMFRDGDGVAYLGNDGTMRMVTDPENVLKGDACWGFSHEVGHVMQMRPMTWGGMTEVSNNIFSLQAAAKTGNE 360
       701 elnSDFNFRIMPMVKNLTggaFMNAHSGVIGIRPG--NQNCIV---GADM---GWGTMHELGHNFDTSGRT---IAEVTNNIMPLYFESLNRTQ 783

       361 SRLKRQGSYDKARKEIIEG----EIAYLQS--KDVFNKLVPLWQLHLYFTKNGHPDFYPDVMEYLRNNAGNYGGNDTVKYQFEFVKACCDVTKT 448
       784 TRITDQNIWENNTYPKVGLddysNNKLYNTsdSTHLAQLAPLWQLYLYDN-----TFYGKFEQQFRANNYGNK--TREDIYKSWVVAASNAMQL 870

       449 DLTDFFEKWGFFKPGKFHIGDYAQYDFNVTPEMVEETKKWIAGKGYPKPETDITELSE 506
       871 DLTEFFARHGIR--------------------INDEVAQEISSK-YEKPDKKIYYLND 907


AM0627  21 ANTPEHIGNDLKLFKDSSCTSLKPDVKNTSAFQSDAMKELATKILAGHYKPDYLYAEYRALPSPRQTGKNLRIGDGFSKYDNMTGVYLEK-GRH 113
  IMPa 437                                               GSEE--TLTLTLPSAQ---------------gFTAIGRMAAPgKRL 465

       114 VVLVGKTEGQEISLLLPNLM--RKPA-EGVQPTKDPNGWGLHKKQ-IPLKEG-INIIDVETPANAYISYF-TEDAGKAPKIPVHFVTGKANGYF 201
       466 SIRIEDAGQASLAVGLNTQrigSTRLwNTRQ---YDRP-rfLKSPdIKLQANqSVALVSPYGGLLQLVYSgATPGQ---TVTVKVTGAASQPFL 552

       202 DTT----RGDTNKDWVRLLDQAVSPIMDARGKYIQVAYPVEFLKKFTK----DRGTELINAYDK-LIGIQYQLMGLD----------------- 269
       553 DIQpgedSSQAIADFIQALDADKADWLEIRSGSVEVHAKVEKVRGSIDkdygGDVQRFIRELNEvFIDDAYTLAGFAipnqaktpaiqqecaar 646

       270 ---KYGK---IPENRVLARVNFN---YYMFRDGDGVAYLGNDGTMRMVTDPENVLKGDACWGFSHEVGHVMQMRPMT--WGGMTEVSNNIFSLQ 352
       647 gwdCDSEtlhKLPGTQHINVDQYaqcGGGCSG-NPYDQT----WGLN----------pRGWGESHELGHNLQVNRLKvyGGRSGEISNQIFPLH 725

       353 AAAKTG-------NESRLKRQGSYDKARKEIIEGE-----------IAYLQS----KDVFNKLVPLWQLHLYFTKNG-----HPDFYPDVMEYL 420
       726 KDWRVLrefgqnlDDTRV----NYRNAYNL-IVAGraeadplagvyKRLWEDpgtyALNGERMAFYTQWVHYWADLKndplqGWDIWTLLYLHQ 814

       421 RNNA--------------------GNYGGNDTVKYQFEFVKACCDVTKTDLTDFFEKWGFFKPGKFHIGDYAQYDFNVTPEMVEETKKWIAGKG 494
       815 RQVDksdwdankaalgygtyaqrpGNSGDASSTDGNDNLLLGLSWLTQRDQRPTFALWGIR--------------------tSAAAQAQVAAYG 888

       495 YPKPETDITELSE 506
       889 FAEQP-AFFYANN 900
```

Figure S4. Sequence alignments between PF13402 proteases. Sequence alignments of PF13402 proteases with AM0627 for structurally similar regions identified using the DALI server. Conserved residues are colored based on side chain chemistry (green: nonpolar, pink: polar, orange: aromatic, blue: acidic, red: basic).

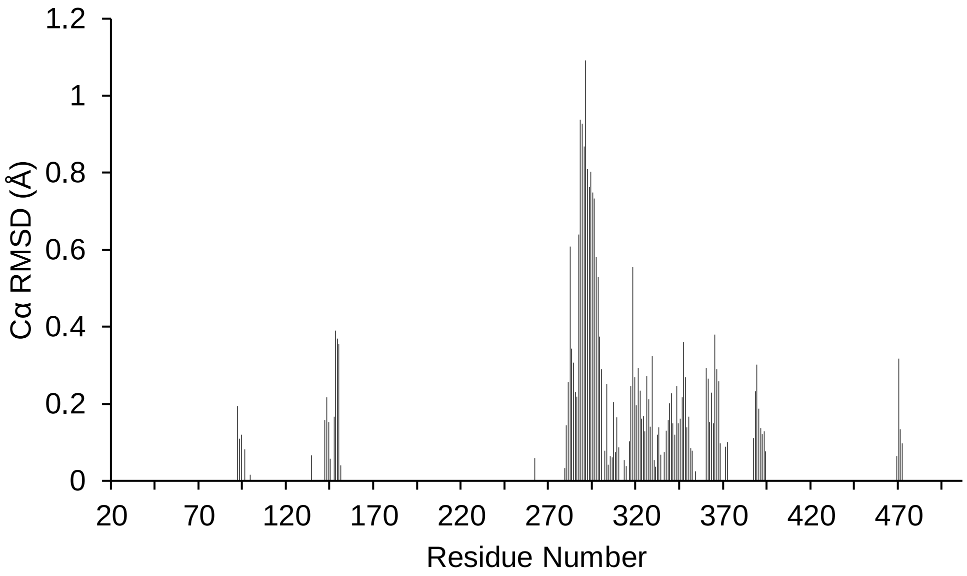

Figure S5. Alpha carbon RMSD values. Root-mean-square deviation (RMSD) values for the alpha carbons of the AM0627 crystal structure and final peptide-docked model structure.

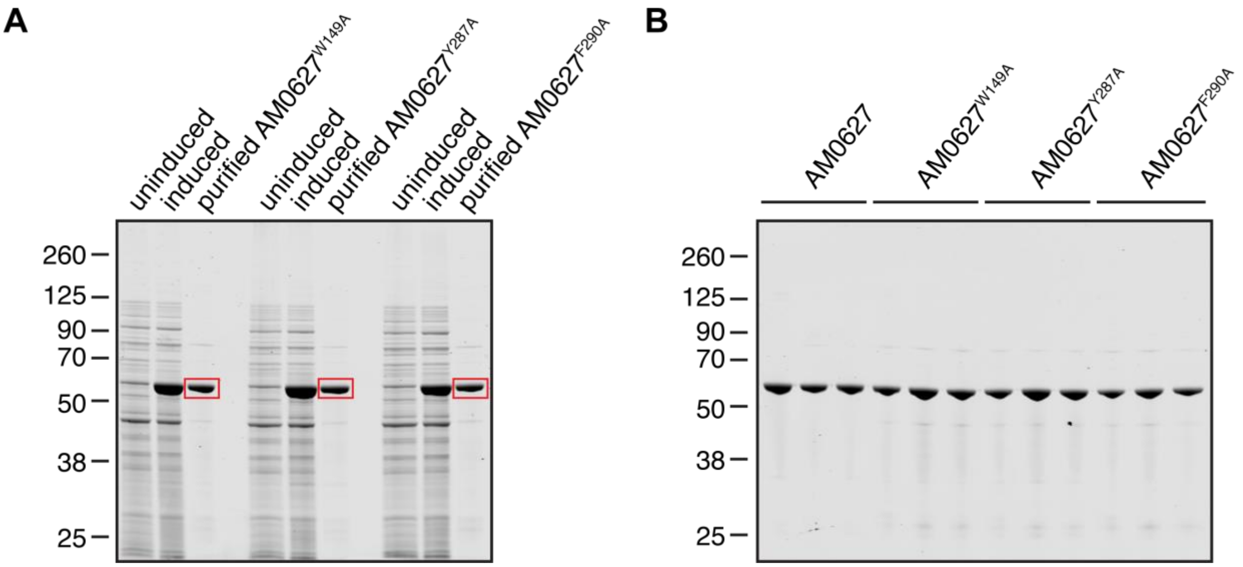

Figure S6. Purification of AM0627 point mutants. *A*, SDS-PAGE analysis of uninduced lysate, induced lysate, and the purified product visualized using Coomassie stain. Protein bands corresponding to each point mutant are denoted by red boxes. *B*, SDS-PAGE analysis of three independent purifications of each variant visualized by Coomassie stain.

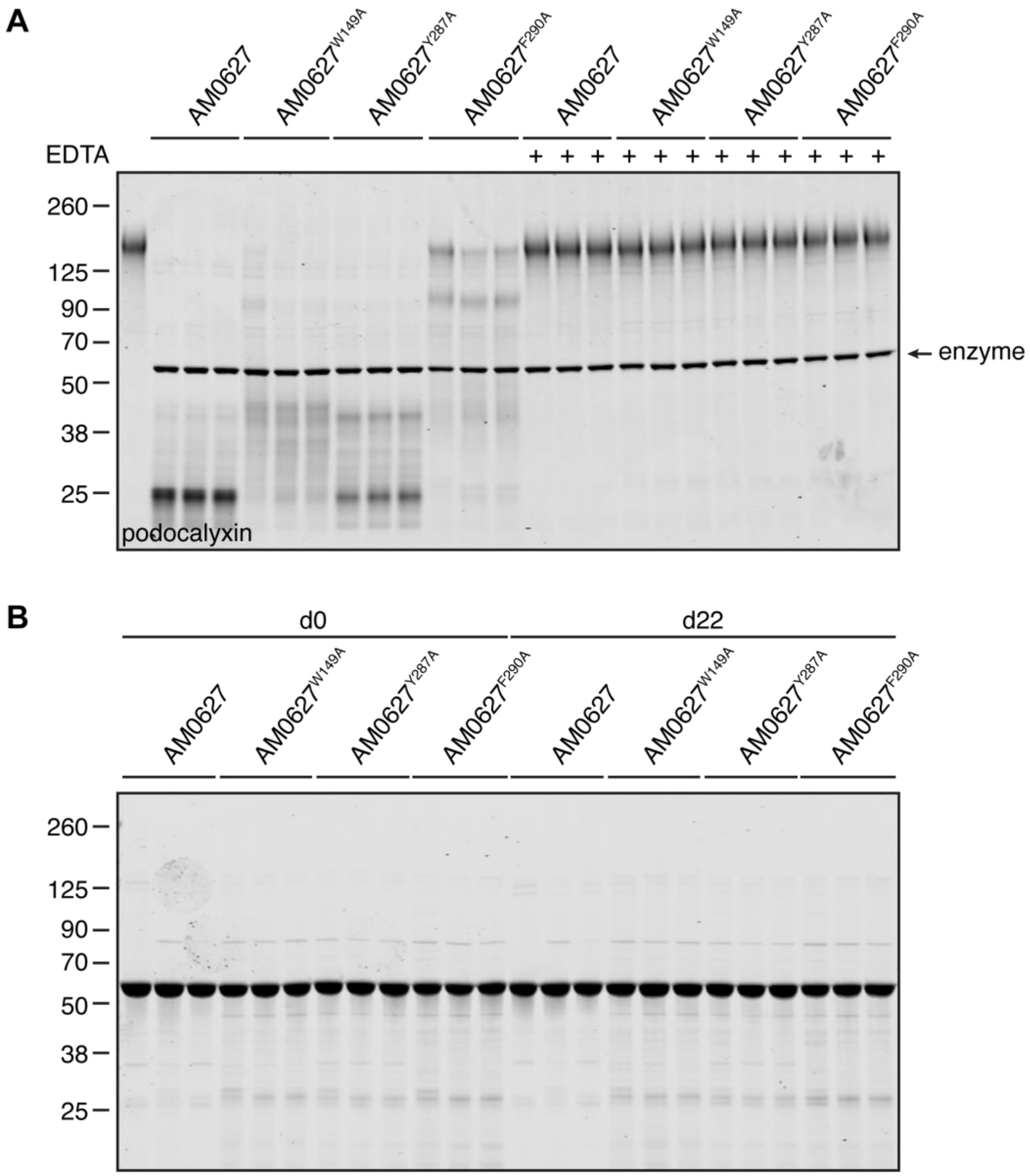

Figure S7. Consistency of activity, metal dependence, and stability between independent AM0627 preparations. *A*, podocalyxin was incubated with 500 nM AM0627 or AM0627 point mutants ± 25 mM EDTA for 22 h at 37 ºC. Digests were separated by SDS-PAGE and visualized using Coomassie stain. *B*, SDS-PAGE analysis of variant preparations at day 0 (d0) and post storage at 4 ºC for 22 days (d22) in PBS.

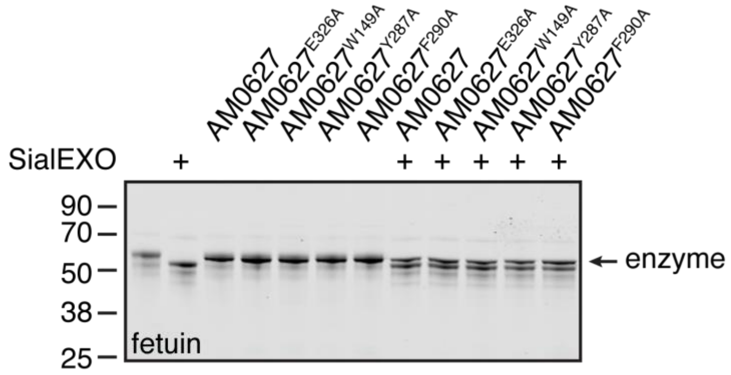

Figure S8. Fetuin digests at high E:S. Fetuin was incubated with 1 µM AM0627 or AM0627 point mutants ± 1 U SialEXO for 22 h at 37 ºC. Digests were separated by SDS-PAGE and visualized using Coomassie stain.

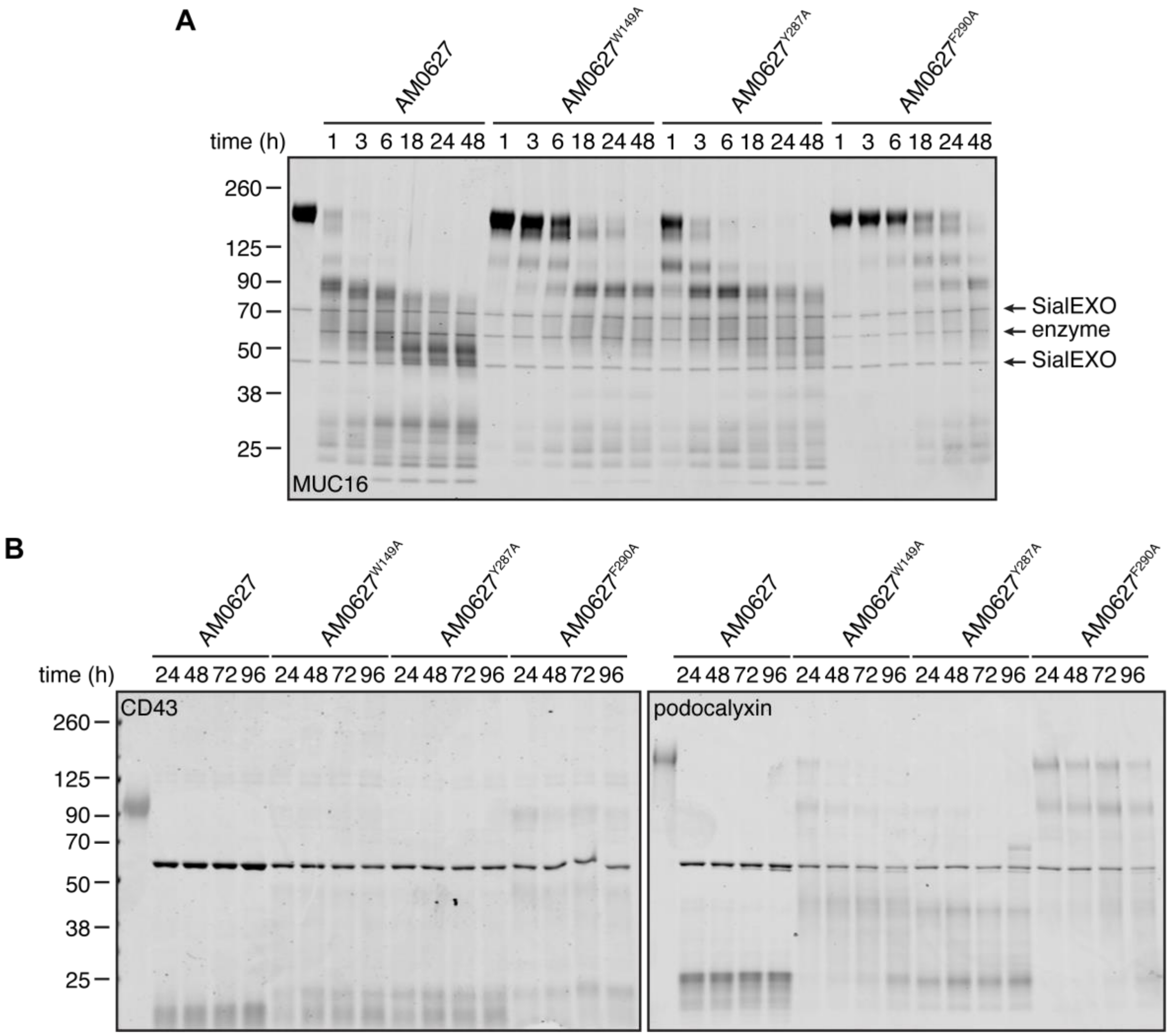

Figure S9. Digestion kinetics. *A*, recombinant MUC16 (0.5 µg) was incubated with 50 nM AM0627 or AM0627 point mutants + 0.5 U SialEXO for the indicated times at 37 ºC. Digests were separated by SDS-PAGE and visualized using Coomassie stain. *B*, recombinant CD43 and podocalyxin (1 µg) were incubated with AM0627 or AM0627 point mutants at a 1:3 enzyme:substrate molar ratio for the indicated times at 37 ºC. Digests were separated by SDS-PAGE and visualized using Coomassie stain.

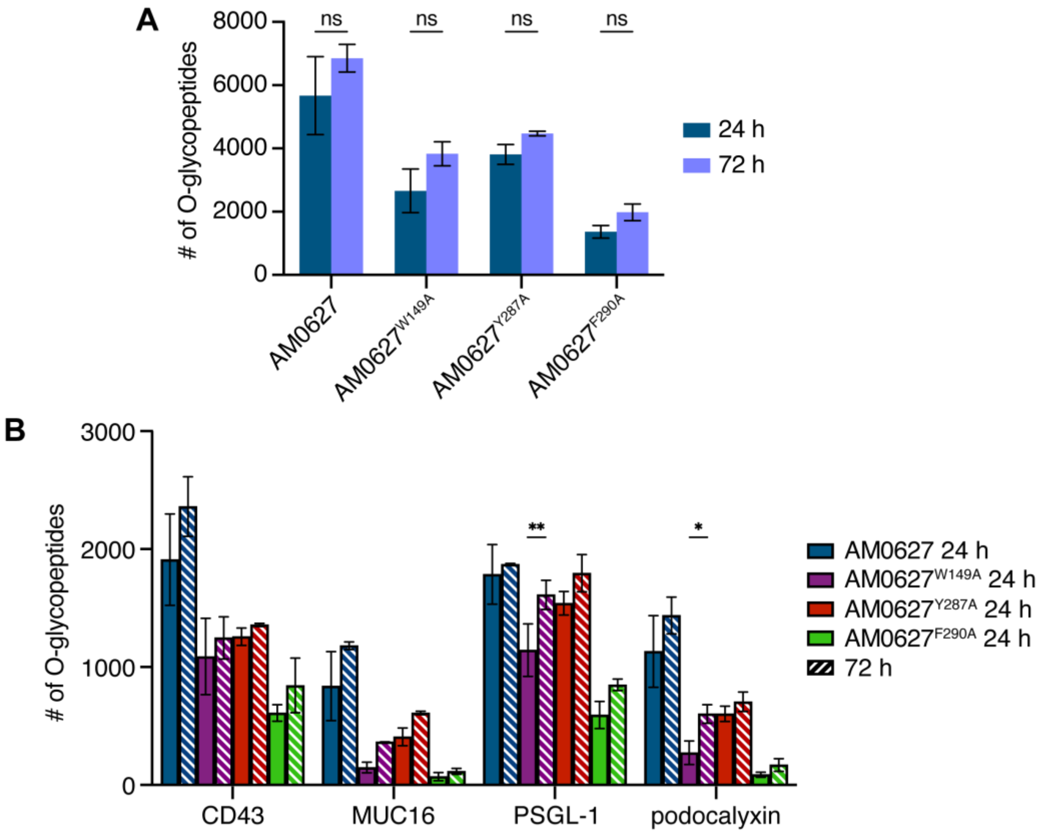

Figure S10. O-glycopeptide counts. *A* and *B*, recombinant glycoproteins (3 µg) were digested with AM0627 or AM0627 mutants at a 1:3 enzyme-to-substrate ratio for 24 or 72 h, de-N-glycosylated, trypsinized, and subjected to mass spectrometry analysis. The total number of O-glycopeptides overall (*A*) and by substrate (*B*) are shown for each enzyme. Data are mean ± s.d. (n = 2). p-values were determined by two-way ANOVA with Bonferroni correction. *$p < 0.05$, **$p < 0.005$.

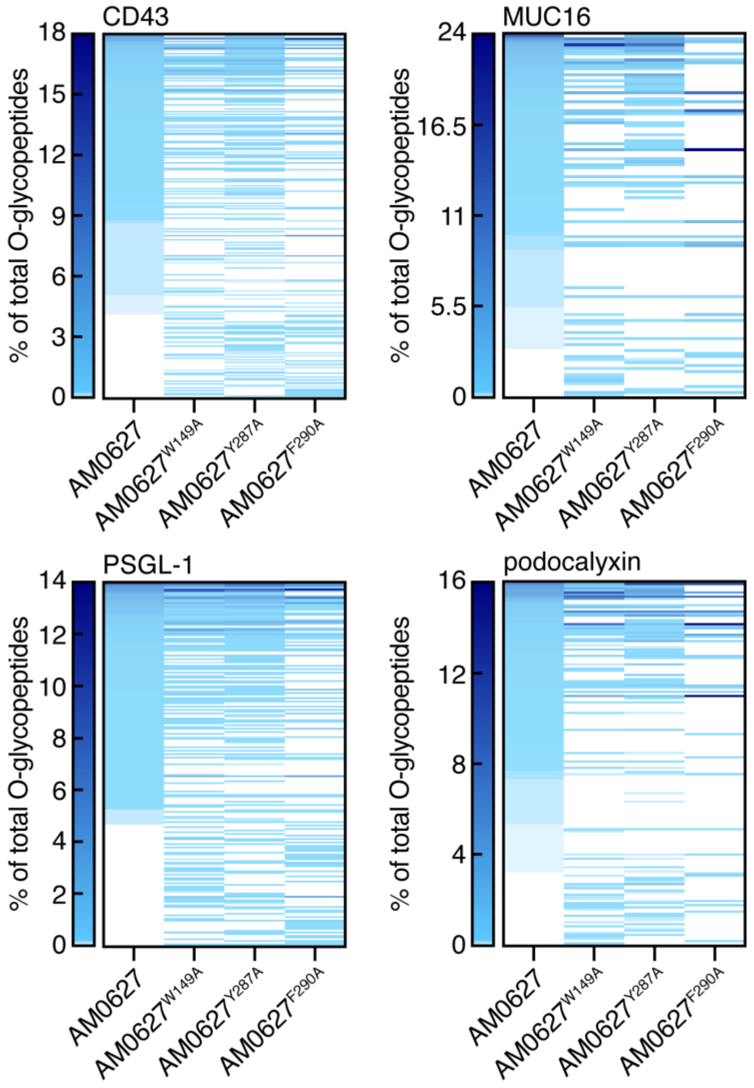

Figure S11. O-glycopeptide sequence heat maps for 72-h digests. Heat maps depicting the frequency of each O-glycopeptide sequence normalized to the total number of substrate O-glycopeptides generated by the enzyme after 72 h. Peptide sequences are ordered from highest to lowest frequency (top to bottom) for AM0627. Specific sequences and counts are listed in Dataset S3.

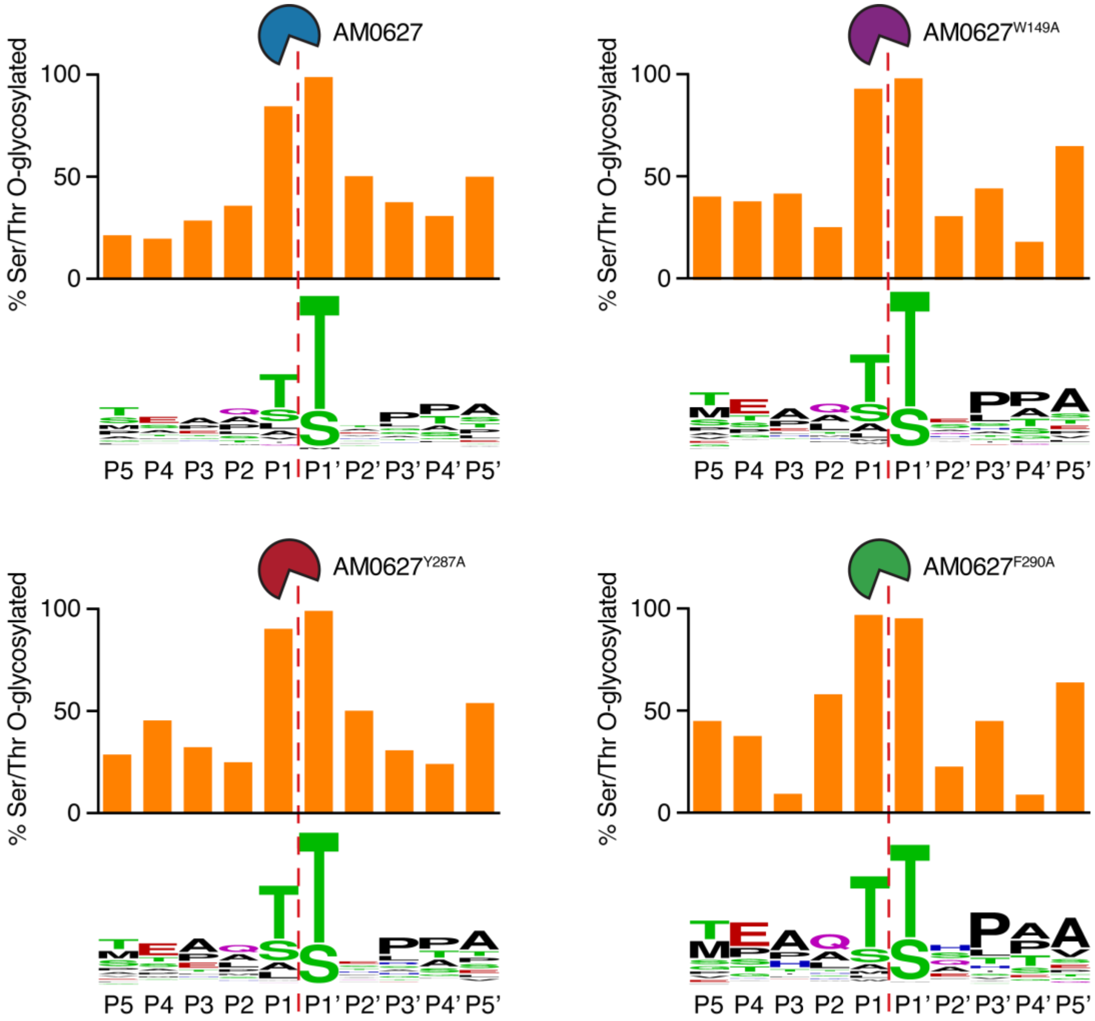

Figure S12. Cleavage motifs. Cleavage motifs determined using O-glycopeptides from 24-h digests. Peptides were used as input for weblogo.berkeley.edu (±5 residues from the site of cleavage). The percent of O-glycosylated serine (Ser) and threonine (Thr) residues was determined by counting the number of modified residues at a given position relative to the total number of serine and threonine residues.

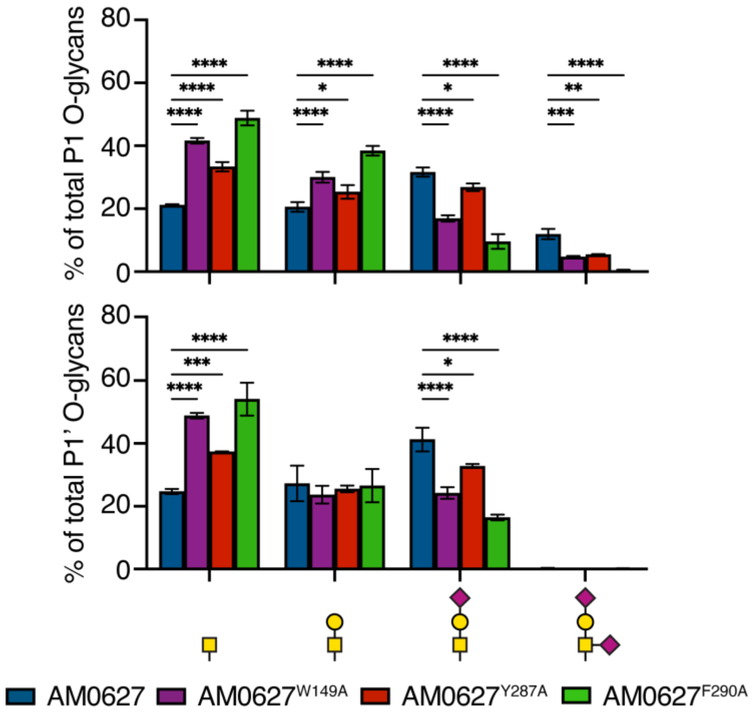

Figure S13. O-glycan occurrences for 72-h digests. Quantification of O-glycan occurrences at P1 (*top*) and P1' (*bottom*) for 72-h digests. Data are mean ± s.d. (n = 2). p-values were determined by two-way ANOVA with Dunnett correction. *p < 0.05, **p < 0.005, ***p < 0.0005, ****p < 0.0001.

Table S3. Cloning primers

Primer	Sequence
AM0627^{W149A} for	5’-CCCCAACGGCGCGGGATTGCATAAAAA-3’
AM0627^{W149A} rev	5’-TCTTTTGTGGGCTGCACT-3’
AM0627^{Y287A} for	5’-GAACTTCAACGCCTACATGTTCCGCGACGGAGACGGAGTCGCC-3’
AM0627^{Y287A} rev	5’-ACGCGGGCCAGGACGCGG-3’
AM0627^{F290A} for	5’-CTACTACATGGCCCGCGACGGAGAC-3’
AM0627^{F290A} rev	5’-TTGAAGTTCACGCGGGCC-3’

Table S4. Glycan compositions used for O-glycoproteomics search

HexNAc(1)	HexNAc(2)Hex(2)Fuc(1)NeuAc(2)
HexNAc(1)Hex(1)	HexNAc(1)Hex(1)NeuGc(1)
HexNAc(1)NeuAc(1)	HexNAc(1)Hex(1)NeuAc(1)NeuGc(1)
HexNAc(2)Hex(1)	HexNAc(1)Hex(1)NeuGc(2)
HexNAc(1)Hex(1)NeuAc(1)	HexNAc(2)Hex(2)NeuGc(1)
HexNAc(1)Hex(1)NeuAc(2)	HexNAc(2)Hex(2)NeuGc(2)
HexNAc(2)Hex(2)NeuAc(1)	HexNAc(2)Hex(2)NeuAc(1)NeuGc(1)
HexNAc(2)Hex(2)NeuAc(2)	HexNAc(2)Hex(1)NeuGc(1)
HexNAc(2)Hex(2)	HexNAc(2)Hex(2)Fuc(1)NeuGc(1)
HexNAc(2)Hex(1)NeuAc(1)	HexNAc(2)Hex(2)Fuc(1)NeuGc(2)
HexNAc(2)Hex(2)Fuc(1)NeuAc(1)	HexNAc(2)Hex(2)Fuc(1)NeuAc(1)NeuGc(1)
